# Semisynthesis and Antitumour Evaluation of Natural Derivatives from *ent*-Kaurene *ent*-15α-Angeloyloxykaur-l6-en-3β-ol Isolated from *Distichoselinum tenuifolium*

**DOI:** 10.3390/ijms252313222

**Published:** 2024-12-09

**Authors:** Yass K. Yasser, Daniel Gil, Houda Zentar, María Jesús Durán-Peña, Belen Prados-Lopez, Jorge Juárez-Moreno, José Manuel Botubol-Ares, Ali Haidour, Juan Sainz, Antonio Fernández, Ramón Alvarez-Manzaneda, Rachid Chahboun, Fernando J. Reyes-Zurita

**Affiliations:** 1 Department of Biochemistry and Molecular Biology I, Faculty of Sciences, University of Granada, 18071 Granada, Spain; yass75@correo.ugr.es (Y.K.Y.); zentarhouda@correo.ugr.es (H.Z.); bprados@ucm.es (B.P.-L.); jorgejmx@correo.ugr.es (J.J.-M.); jsainz@ugr.es (J.S.); 2 Department of Organic Chemistry, Faculty of Sciences, University of Granada, 18071 Granada, Spain; da.gilsal@alum.uca.es (D.G.); ahaidour@ugr.es (A.H.); ajfvargas@ugr.es (A.F.); 3 Department of Organic Chemistry, Faculty of Sciences, Campus Universitario Río San Pedro, University of Cádiz, 11510 Puerto Real, Spain; mariajesus.duran@uca.es (M.J.D.-P.); josemanuel.botubol@uca.es (J.M.B.-A.); 4 Genomic Oncology Area, GENYO, Centre for Genomics and Oncological Research: Pfizer/University of Granada/Andalusian Regional Government, PTS, 18016 Granada, Spain; 5 CIBER Epidemiología y Salud Pública (CIBERESP), 28029 Madrid, Spain; 6 Instituto de Investigación Biosanitaria IBs.Granada, 18012 Granada, Spain; 7 Área de Química Orgánica, Departamento de Química y Física, Universidad de Almería, 04120 Almería, Spain; ralvarez@ual.es

**Keywords:** *Ent*-kaurenes, antitumour activity, apoptosis, cell cycle, mitochondrial membrane potential

## Abstract

Two natural *ent*-kaurene diterpenoids, *ent*-15α-angeloyloxykaur-16-en-3β-ol (**7**) and *ent*-15α-angeloyloxykaur-16-en-3β,9-diol (**8**), were extracted from the aerial parts of *Distichoselinum tenuifolium*, and six new derivatives were synthesised from compound (**7**). The antitumour properties of these natural and derivative *ent*-kaurenes (**2, 7**, **9**–**13**) were evaluated in three cancer cell lines: HT29 (colon cancer), HepG2 (hepatocellular carcinoma), and B16-F10 (murine melanoma). Among them, the synthesised *ent*-kaurene (**13**) containing an exomethylene–cyclopentanone moiety showed the strongest antiproliferative effects in all cell lines tested, with significantly lower IC_50_ values around 2.5 μM. Compounds **13** and **12**, together with their precursor (**7**), were selected for further comparative cytometric and microscopic analyses. Cell cycle studies revealed that derivatives **12** and **13** exhibited promising cytostatic activity by inducing selective G2/M phase arrest, particularly effective in HT29 and HepG2 cells. Conversely, precursor (**7**) showed no significant effect on B16-F10 cell cycle distribution. The Annexin V-FITC/PI double staining assay confirmed the robust apoptotic effects of compounds (**7**), **12** and **13**, with compound 13 inducing up to 99% total apoptosis and exhibiting significant apoptotic activity in all cell lines tested. These apoptotic effects were closely linked to mitochondrial dysfunction, as evidenced by a marked loss of mitochondrial membrane potential and reduced Rh123 fluorescence in treated cells, thereby activating the intrinsic apoptotic pathway. These findings highlight the critical role of mitochondrial disruption in the cytotoxic mechanisms of these *ent*-kaurenes and underscore their potential as promising anticancer agents.

## 1. Introduction

Diterpenes are an abundant natural product in plant species and constitute a source of important structural variety. As secondary metabolites, they are not considered essential for the life of the species that contain them; however, they play an important role in their survival. The *ent*-kauranoids are a relatively small group of diterpenoids characterised by a tetracyclic structure and are generally found in higher plants. These diterpenes exhibit interesting pharmacological activities, including anticancer, antimicrobial, and anti-inflammatory properties [1,2,3]. Since their initial discovery in 1961, approximately 1300 *ent*-kaurene derivatives have been identified across various plant families such as Apiaceae, Asteraceae, Annonaceae, Lamiaceae, Euphorbiaceae, Jungermanniaceae, and Chrysobalanaceae. Among these, the genus *Isodon* stands out for its abundant production of structurally distinct and bioactive *ent*-kaurenes, including oridonin, a potent tetracyclic diterpenoid that has progressed to phase I clinical trials due to its promising anticancer activity against diverse malignancies such as acute myeloid leukaemia and pancreatic, colorectal, and liver cancers [4]. Among the four subgroups of *ent*-kaurane diterpenoids, non-oxygenated *ent*-kaurenes, oxygenated *ent*-kaurenes, and 8,9-seco-*ent*-kauranoids can be highlighted [2,5].

**Figure 1:**
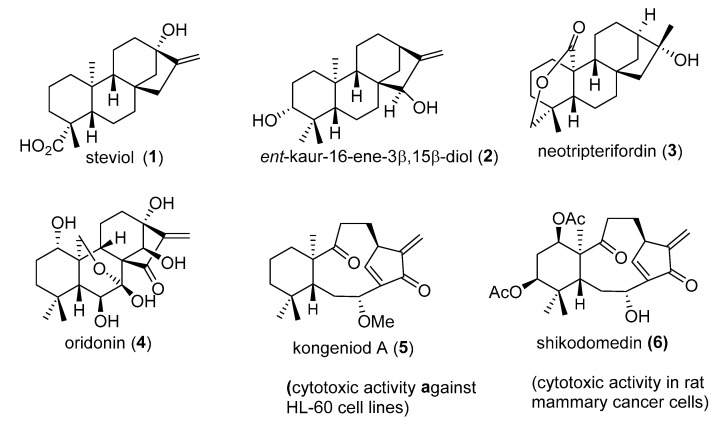
Some examples of *ent*-kaurane types.

Aglycone steviol (**1**) is an example of non-oxygenated *ent*-kaurenes isolated from the species *Stevia rebaudiana*, which is produced via enzymatic hydrolysis of the steviol glycoside stevioside. Steviol glycoside extracts are natural low-calorie sweeteners since they are up to 300 times sweeter than sucrose; they are widely used sweeteners in many countries, including the United States. *ent*-Kaur-16-ene-3b,15b-diol (**2**) was first isolated from the Japanese species *Jungermunnia vulcanicola* [6], and later was extracted from *Distichoselinum tenuifolium* [7] and isolated from the roots of *Gelonium multiflorum* [8]. Recently, diol **2** was isolated from *Erythroxylum bezerrae,* and its anti-inflammatory activity was evaluated. It was found to exhibit significant nitric oxide (NO)-inhibitory activity with an IC_50 NO_ value of 3.76 mM [9]. Moreover, Neotripterifordin (**3**), which was extracted from the Chinese medicinal plant *Tripterygium wilfordii* [10,11], inhibited HIV replication in H9 human lymphocyte cells with an EC_50_ of 25 nM. Oridonin (**4**), which was isolated from the ethanolic extract of *Isodon japonica*, shows strong specific antibacterial activity against Gram-positive *Bacillus subtilis* bacteria with a potent and selective antitumor activity [12,13,14]. This high biological activity is mainly attributed to the conjugate bond system, which facilitates Michael-type nucleophilic addition reactions involving, for example, cysteine residues. 8,9-seco-*ent*-kauranoids are tricyclic diterponoids characterised by an unusual cyclohexyl-fused bicyclo [7.2.1]dodecane framework. This group of *ent*-kaurenes exhibits activities such as cytotoxicity against HeLa, KB, and FM 3A/B cell lines, antimycobacterial or antimalarial activities, antibacterial activity against *Staphylococcus aureus*, and apoptosis in SW480 cells [15,16,17,18]. Kongeniod A (**5**), which was extracted from the aerial parts of *Croton kongensis* [15], and shikodomedin (**6**), which was isolated from *Rabdosia shikokiana* var. *intermedia* [16], are two examples of the 8,9-seco-kauranoids group (Figure 1).

Within the Apiaceae family, *Distichoselinum tenuifolium*, a monotypic species endemic to Spain, has emerged as an intriguing source of structurally unique *ent*-kaurene diterpenoids [7,19]. Recent studies have identified these compounds as potent cytotoxic agents against cancer cell lines, with an activity influenced by specific structural features. These structural elements were linked to their mechanism of action, which involves both cytostatic and cytotoxic effects, as exemplified by compounds like pharicin A and kaurenoic acid [20,21].

**Figure 2:**
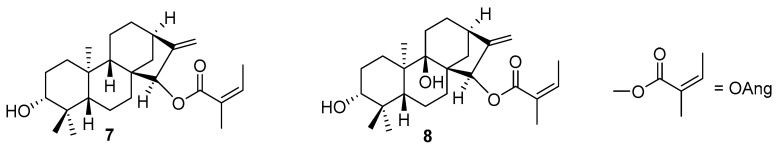
Representative *ent*-kaurane from *Distichoselinium tenuifolium*.

Grande’s research group conducted a study of the hexane extract of the roots of *Distichoselinium tenuifolium*, a botanic species traditionally used for the treatment of dermatitis and skin infections [22]. From this plant, six compounds with *an ent*-kauran diterpene skeleton were isolated, with *ent*-15a-Angeloyloxykaur-l6-en-3b-ol (**7**) and *ent*-15a-angeloyloxykaur- 16-ene- 3b, 9-diol (**8**) beign the most important products [7,19,23,24] (Figure 2).

In this work, we focus on the anticancer potential of *ent*-kaurene diterpenoids derived from *D. tenuifolium*. Specifically, we explore their mechanisms of action, their efficacy across various cancer types, and how structural modifications can enhance their therapeutic potential. The main *ent*-kaurenes in the *Distichoselinium tenuifolium* were isolated and two natural compounds and some derivatives were prepared from **7**. The antiproliferative activities of the isolated natural products and the different derivatives that were prepared were evaluated in colorectal (HT29), liver (HepG2), and melanoma (B16-F10) cancer cell lines. A comprehensive analysis combining cytotoxicity assays, cell cycle studies, apoptosis evaluations, and mitochondrial membrane potential (MMP) assessments provided detailed mechanistic insights into their mode of action.

## 2. Results

### 2.1. Chemistry

*Distichoselinum tenuifolium* was collected from the northern part of Granada (South Spain). The hexane-soluble fraction of the methanolic extract was fractionated and purified using silica gel column chromatography to isolate *ent*-kaurenes **7** and **8**, in which compound **7** represents more than 70% of the extract. The NMR data of **7** (Tables S1 and S2, see the ESI) pointed to a kaurane skeleton with an angeloyloxy group and a hydroxyl group attached at C-15 and C-3, respectively, determined using 2D NMR data. Their ^1^H and ^13^C NMR data were in accordance with those previously described for *ent*-15α-angeloyloxykaur-16-en-3β-ol (**7**) [7,19]. However, limited NMR data were provided in that report, and the relative configuration of the angeloyloxy and hydroxyl groups were tentatively assigned on the basis of the calculated ^13^C NMR chemical shifts. The one-dimensionalNOE correlations between H-3/H-5/H-1β/H_3_-18 indicated that they all are β-oriented, while the correlations between H_3_-19/H-14α, H-15/H_2_-17/H-14α/H-7α and H-13α/H_2_-17/H_2_-14 supported their α-orientation (Figure S1, included in the Supplementary Materials). Compound **8** was isolated from the most polar fractions and showed spectroscopic data that were closely structurally related to **7,** with an additional hydroxyl group from a hydroxylated quaternary carbon at δ_C_ 79.2. The position of this group was assigned to C-9 because of the correlations between the protons δ_H_ 2.12 and 1.54-1.42 (H_2_-11), 1.26 (H-7), 1.39-1.29 (H-14), 3.36 (9-OH) and 1.15 (H_3_-20), and carbon C-9 (δ_C_ 79.2) in the g-HMBC spectrum. Furthermore, the spectroscopic data of **8** were in agreement with those described in the literature for *ent*-15β-angeloyloxykaur-16-en-3β,9α-diol (**8**) [7]. The NMR data were recorded in CDCl_3_ to collate them with the literature. However, due to the stability issues in CDCl_3_, NMR in acetone-*d_6_* was used to acquire the full NMR characterisation of **8**. The relative configuration of **8** was confirmed based on the correlations observed in the NOESY spectrum. The correlations of H-3/H-5/H_3_-18/H-7β indicated that these protons are β-oriented, whereas the correlations of H_3_-20/H_3_-19/H-14a/H-12α/H-11α and H-15/H-7α/H-14b/H-17a were used to place these protons on the opposite face of the molecule (Figure S2, included in the Supplementary Materials).

**Scheme 1:**
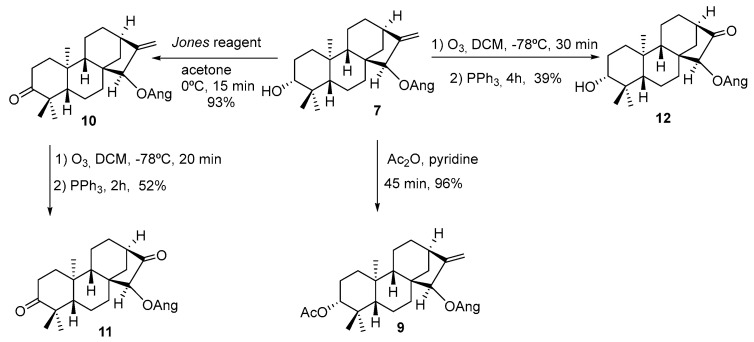
Preparation of some derivatives of **7**.

Next, some derivatives were prepared from *ent*-kaurene **7**. First, compound **6** was acetylated with Ac_2_O/pyridine to create compound **9**. It should be noted that this reaction was described previously [19]. Next, the treatment of **7** with *Jones* reagent in acetone provided ketone **10**, and the oxidation reaction of **7** to **10** was also achieved by Grande’s group using the reagent PCC in CHCl_3_ [19]. The selective oxidation of **10** with ozone was conducted to obtain diketone 11. Compound **7** was directly oxidised with ozone to afford hydroxyketone **12**, which was obtained with a moderate yield 39% (Scheme 1).

**Scheme 2:**
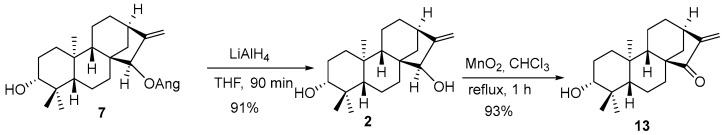
Semisynthesis of natural products *ent*-kaurene-3b,15a-diol (**2**) and *ent*-3b-hydroxykaurene-15-one (**13**) from **7**.

Furthermore, attempts to remove the angelate group from **7** using KOH/MeOH at room temperature or reflux was unsuccessful. However, diol **2** was obtained by reducing **7** with LiAlH_4_ in THF after 90 min at room temperature. The spectroscopic data for **2** are in agreement with those described in the literature [6,7,8]. Moreover, the selective oxidation of diol **2** with MnO_2_ at reflux in CHCl_3_ led to the production of hydroxyenone **13** with a 93% yield. The spectroscopic data for compound **13** were identical to those reported in the literature [6] (Scheme 2). In summary, the preparation of compounds **2** and **13** was performed for the first time from the compound *ent*-15a-angeloyloxikaur-16-en-3b-ol (**7**) very abundant in the species *Distichoselinum tenuifolium*. The structure of compound **7**, which was identified in several studies, allows us to establish the absolute stereochemistry of the two synthesised natural compounds.

### 2.2. Anticancer Activity

#### 2.2.1. Cell Viability

The evaluation of cell viability is fundamental in determining the cytotoxic potential of new compounds against cancer cells. In this study, both synthesised and naturally derived *ent*-kaurenes (compounds **2**, **7**, **8**, **9**, **11**, **12**, **13**) were evaluated for their in vitro antiproliferative activity against three tumour cell lines: human colon adenocarcinoma (HT29, ECACC No. 9172201; ATCC No. HTB-38), human hepatocarcinoma (HepG2, ECACC No. 85011430), and murine melanoma (B16-F10, ATCC No. CRL-6475). The MTT (3-(4,5-dimethylthiazol-2-yl)-2,5-diphenyltetrazolium bromide) colorimetric assay was used, a standard method for assessing cell metabolic activity, which directly correlates with cell viability. This assay measures the reduction in MTT dye to create formazan via the mitochondria in viable cells.

To assess the potency of these *ent*-kaurenes, each product was incubated in different cell lines at concentrations ranging from 0 to 100 µg/mL. IC_20_, IC_50_, and IC_80_ values (the concentrations required to inhibit cell growth by 20%, 50% and 80%, respectively) were calculated for each compound, as shown in Table 1.

**Figure 3:**
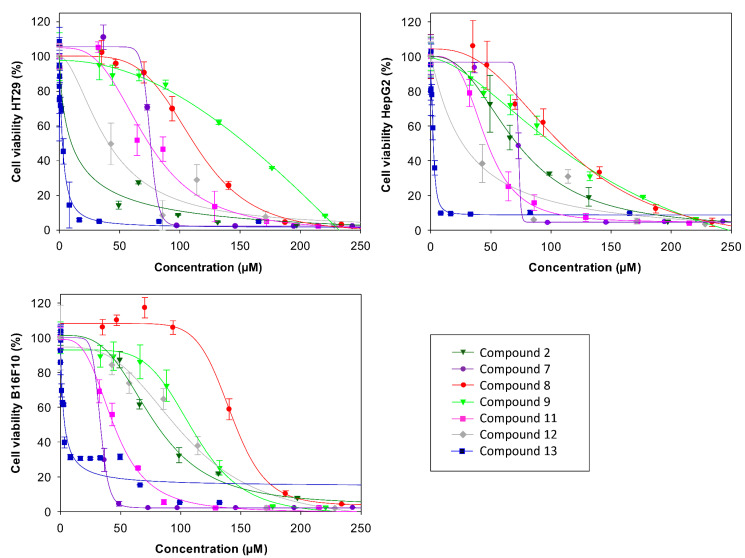
Sigmoidal cell viability curves (or cytotoxicity) for kaurene derivatives in the three cell lines studied.

**Figure 4:**
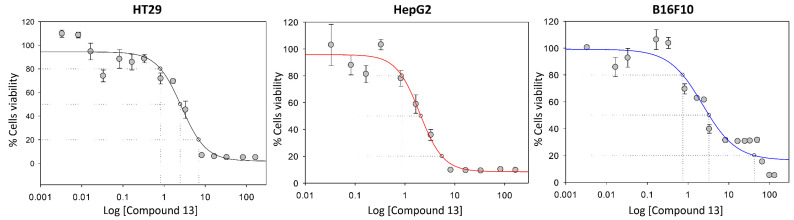
Dose–response curves of *ent*-kaurene **13** in the three cancer cell lines. Relative viability percentages with respect to the untreated control were plotted against the logarithm of the concentration of each tested compound. Each data point represents the mean ± standard deviation from three replicates per concentration.

These results show that all tested compounds exhibited dose-dependent cytotoxicity in the three cancer cell lines, as shown in Figure 3. Among the tested *ent*-kaurenes, compound **13** showed the strongest antiproliferative effects, with significantly lower IC_50_ values in HT29, HepG2, and B16-F10 cells: 2.71 ± 0.23 μM, 2.12 ± 0.23 μM, and 2.65 ± 0.13 μM, respectively. These low IC_50_ values suggest that compound **13** may be a particularly promising candidate for further study due to its potent cytotoxic activity at relatively low concentrations, as illustrated by the sigmoidal dose–response curves in Figure 4.

In addition, compounds **2**, **7** and **12** showed selectivity against HT29, B16-F10, and HepG2 cell lines, with IC_50_ values of 10.08 ± 2.69 μM for compound **2** in HT29 cells, 32.43 ± 3.28 μM for compound **7** in B16-F10 cells, and 24.43 ± 16.35 μM for compound **12** in HepG2 cells. Notably, compound **13** exhibited higher toxicity against HepG2 cells, with an IC_50_ value almost 35 times lower than that of its precursor **7**, highlighting a potential specificity for hepatocarcinoma cells. Similarly, compound **13** was 12 and 38 times more cytotoxic than its precursor 7 against B16-F10 and HT29 cells, respectively. This significant difference in cytotoxicity suggests that compound **13** may exert its effects through different mechanisms of action or may exhibit enhanced uptake or metabolic activation in certain cell types.

Other kaurenes, including compounds **8**, **9**, and **11**, showed moderate cytotoxic effects in all cell lines, with IC_50_ values ranging from 42.03 μM to 153.23 μM. Although less potent than compound **13**, the variation in the IC_50_ values between cell lines suggests a potential for differential activity that could be explored in further structure–activity relationship (SAR) studies of these *ent*-kaurenes.

On the basis of these results, and in view of their promising cytotoxic activity, natural kaurene **7** and its kaurene derivatives, compounds **12** and **13**, were selected for further cytometric and microscopic analyses. These analyses aimed to characterise the changes in cell cycle progression, the apoptotic mechanisms, and the alterations in cell morphology and mitochondrial membrane potential, which will help elucidate the cellular mechanisms underlying their cytotoxic effects.

#### 2.2.2. Cell Morphology

The cytotoxic effects of *ent*-kaurene **13** on HT29, HepG2, and B16-F10 cells and their morphological changes were confirmed and evaluated via inverted microscopy. The results of these observations reveal a progressive sequence of morphological changes that correlate with dose-dependent cytotoxic effects across the three cell lines. At IC_50_ concentrations, compound **13**, together with other active derivatives, such as compounds **2**, **7**, and **12**, showed a significant inhibition of cell proliferation. The results of the compound **13** were consistent after 72 h of treatment in the three treated lines, showing distinct apoptotic characteristics.

**Figure 5:**
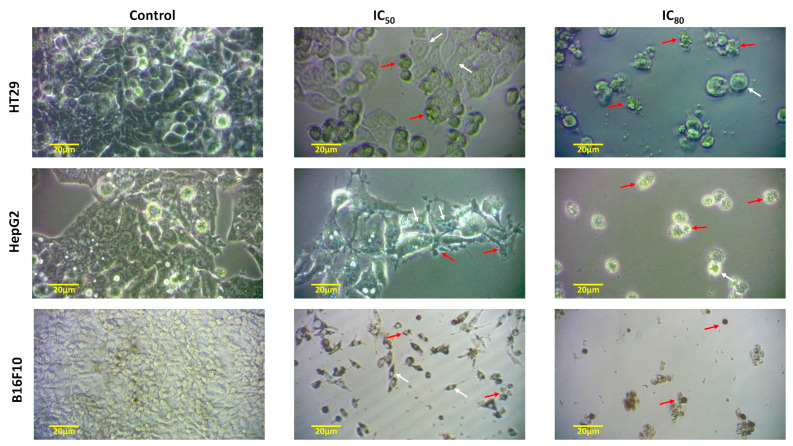
Light microscopy images from the contrast phase of control cells (untreated) and cells treated with IC_50_ and IC_80_ concentrations of *ent*-kaurene **13** (white arrows indicate live cells; red arrows indicate apoptotic or dead cells).

Figure 5 provides a clear comparison, showing that untreated control cells retained a large, flattened morphology with strong adhesion to the culture surface and a generally uniform, rounded shape with visible membrane integrity. Following treatment with IC_50_ concentrations of compound **13**, cells of all lines (HT29, HepG2, and B16-F10) exhibited marked changes in morphology. Cells appeared smaller and more rounded, with a scattered distribution suggesting reduced intercellular adhesion. In addition, cell shrinkage was evident, indicating an early apoptotic response characterised by cytoplasmic condensation and loss of typical membrane morphology.

As the concentration increased to IC_80_ levels, more pronounced morphological features of cell death were observed. A marked increase in apoptotic cells was observed, and membrane blebbing and rupture of the cell membrane were identified, leading to the formation of apoptotic bodies and cellular debris. These effects, observed at high concentrations, suggest that compound **13** induces extensive cell death beyond the initial cytostatic effects, progressing to apoptosis, cell degradation, and disintegration.

The contrasting morphologies (control versus treated cells) shown in Figure 5 emphasise this differential response, as untreated cells showed adherence, a uniform shape and intact cell membranes, in contrast to the rounded, fragmented, and scattered morphology observed in treated cells. This clear apoptotic response, coupled with mitochondrial membrane alterations, highlights the potential of compound **13** and related *ent*-kaurene derivatives as promising anticancer agents that are capable of inducing cell death in colorectal, liver, and melanoma cancer cell lines.

#### 2.2.3. Induction of Apoptosis

Apoptosis, or programmed cell death, is characterised by a cascade of biochemical events leading to morphological and structural changes, including chromatin condensation, nuclear fragmentation, and cell membrane blebbing. These features culminate in the formation of apoptotic bodies [25]. The induction of apoptosis is a central target in cancer therapy as it allows for the focused elimination of cancer cells via programmed cell death mechanisms. While cell cycle arrest is crucial to halt proliferation, apoptotic pathways provide an additional layer of cytotoxicity that enhances the anticancer effects of therapeutic agents. The majority of currently used anticancer drugs trigger and induce apoptosis as a key target in cancer cells [25]. Therefore, a comprehensive evaluation of the apoptotic potential of compounds **7**, **12**, and **13** was performed on the three cancer cell lines to elucidate their mechanisms of action beyond cell cycle inhibition.

**Figure 6:**
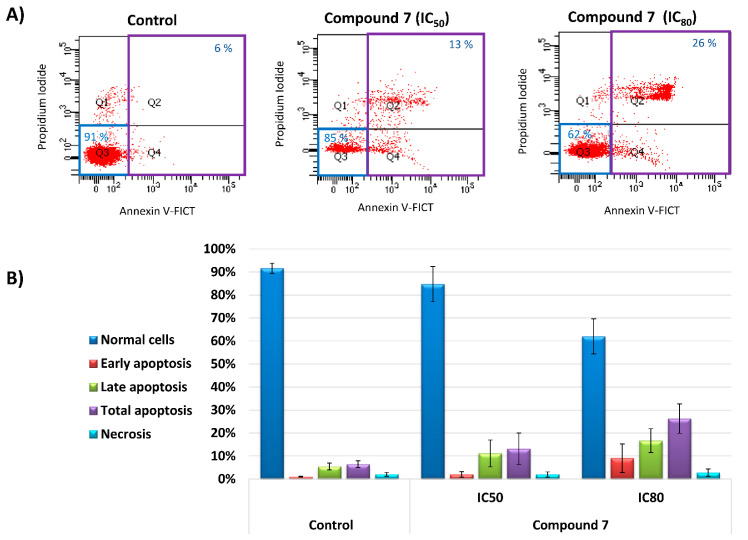
Effects of compound **7** on apoptosis in B16-F10 cells after 72 h treatment, at IC_50_ and IC_80_ concentrations. (**A**) Annexin V/propidium iodide (IP) cytometry plots: Q1, necrotic cells (Annexin V− PI+); Q2, late apoptotic cells (Annexin V+ PI+); Q3, normal cells (Annexin V− PI−); Q4, early apoptotic cells (Annexin V+ PI−); normal cells, blue quadrant; apoptotic cells, purple quadrant. (**B**) Flow cytometric analysis of Annexin V-FITC staining and PI accumulation: normal cells (blue bars), early apoptotic cells (brown bars), late apoptotic cells (green bars), and total apoptotic cells (purple bars). Values represent mean ± SD of three experiments performed in duplicate.

**Figure 7:**
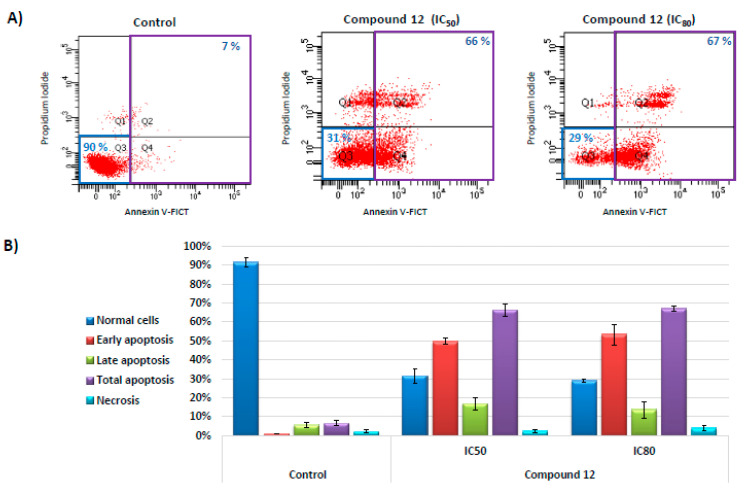
Effects of compound **12** on apoptosis in HepG2 cells after 72 h treatment at IC_50_ and IC_80_ concentrations. (**A**) Annexin V/propidium iodide (IP) cytometry plots. Q1, necrotic cells (Annexin V− PI+); Q2, late apoptotic cells (Annexin V+ PI+); Q3, normal cells (Annexin V− PI−); Q4, early apoptotic cells (Annexin V+ PI−); normal cells, blue quadrant; apoptotic cells, purple quadrant. (**B**): Flow cytometric analysis of Annexin V-FITC staining and PI accumulation: normal cells (blue bars), early apoptotic cells (brown bars), late apoptotic cells (green bars), and total apoptotic cells (purple bars). Values represent mean ± SD of three experiments performed in duplicate.

**Figure 8:**
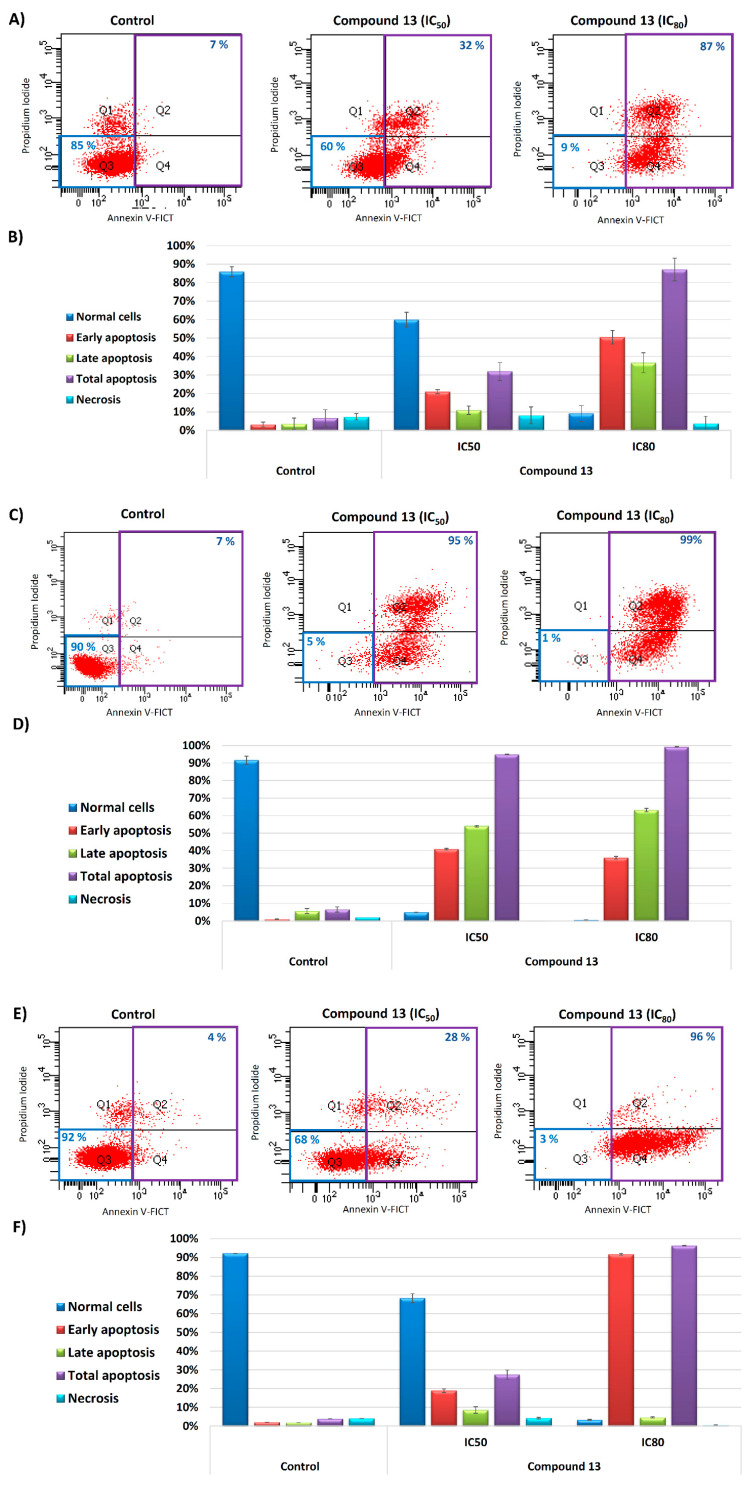
Effects of compound **13** on apoptosis in HT29 cells (**A**,**B**), HepG2 cells (**C**,**D**), and B16-F10 (**E**,**F**) after 72 h treatment at IC_50_ and IC_80_ concentrations. (**A**,**C**,**E**) Annexin V/propidium iodide (IP) cytometry plots; normal cells, blue quadrant; apoptotic cells, purple quadrant. (**B**,**D**,**F**): Flow cytometric analysis of annexin V-FITC staining and PI accumulation. Values represent mean ± SD of three experiments performed in duplicate.

In this context, we further investigated the potential mechanisms underlying the cytotoxic and cytostatic effects of compounds **7**, **12**, and **13** on the three cancer cells using Annexin V-FITC/PI double staining together with a flow-activated cell sorter (FACS). This method allowed for differentiation between early apoptosis, late apoptosis, and necrosis. This approach allowed for the detection of phosphatidylserine (PS) externalisation. In brief, the loss of cytoplasmic membrane asymmetry that occurs during apoptosis causes the translocation of phosphatidylserine (PS) from the inner membrane leaflet to the outer membrane, exposing PS to the external environment, where it is recognised and rapidly phagocytosed by macrophage cells [26]. Annexin V binds specifically to exposed PS, allowing for the identification of early apoptotic cells (Annexin V+/PI−), late apoptotic cells (Annexin V+/PI+), and necrotic cells (Annexin V−/PI+), as shown in Figure 6, Figure 7 and Figure 8. This staining was performed 72 h after treatment at IC_50_ and IC_80_ concentrations, providing insight into concentration-dependent apoptotic responses.

The apoptotic effects of compound **7** on B16-F10 cells are as follows: Exposure to compound **7** resulted in a dose-dependent increase in apoptotic cell populations on the B16-F10 melanoma cell line. Control samples showed 91% normal cells, which decreased to 85% and 62% at IC_50_ and IC_80_ concentrations, respectively. This decrease in normal cells was accompanied by an increase in total apoptosis rates from 6% in control cells to 13% and 26% at IC_50_ and IC_80_, respectively. Notably, early apoptotic cells constituted the majority of the apoptotic population at both concentrations, accounting for 11% and 16% of the total cells, respectively. No significant necrotic activity was observed, suggesting that compound **7** primarily induces apoptosis rather than necrosis in melanoma cells, thereby reducing non-specific cell death (Figure 6).

The apoptotic effects of compound **12** on HepG2 cells were as follows: Compound **12** showed significant apoptotic effects on the HepG2 hepatocarcinoma cell line, with total apoptosis rates reaching 67% at both the early and late stages, even at lower concentrations. Early and late apoptosis accounted for 43% and 24% of the total apoptotic population, respectively, while necrotic cell populations did not exceed 4% in treated samples.

The proportion of normal cells decreased significantly, from 87% in untreated controls to 29% with compound **12** at both IC_50_ and IC_80_ concentrations, indicating a strong induction of programmed cell death at both concentration levels. These results underscore the potent apoptotic induction of compound **12** in hepatocarcinoma cells, suggesting its potential for targeted cytotoxicity against liver tumours (Figure 7).

The apoptotic effects of compound **13** in the three cell lines were as follows: Among the compounds tested, compound **13** exhibited the most pronounced apoptotic effects in all three cell lines (HT29, HepG2, and B16-F10), demonstrating both early and late apoptotic induction in a dose-dependent manner (Figure 8). In HT29 (colon adenocarcinoma) cells, compound **13** produced a significant apoptotic response, achieving a total apoptosis rate of 32% (21% early and 11% late) at the IC_50_ concentration. At the IC_80_ concentration, the apoptotic effects were even more pronounced, with 87% total apoptosis (50% early and 37% late apoptosis), confirming the potential of compound **13** for high-impact cytotoxicity in colon cancer cells. In HepG2 cells, compound **13** induced remarkably high levels of apoptosis, achieving total apoptosis rates of 95% at IC_50_ (41% early; 54% late) and reaching 99% at IC_80_ (36% early and 63% late apoptosis). This exceptional response suggests a particularly potent effect on hepatocarcinoma cells, probably involving a rapid apoptotic response, in agreement with the results found in the cytotoxicity analysis of this compound.

Finally, in the B16-F10 melanoma cell line, compound **13** produced moderate apoptotic rates at the IC_50_ concentration (28% total apoptosis; 9% early, 19% late), which increased dramatically to 96% at the IC_80_ concentration (92% early, 4% late apoptosis), indicating a broad apoptotic response at higher concentrations.

The observed apoptotic responses suggest that compounds **12** and **13** are particularly effective in inducing apoptosis, with concentration-dependent shifts from cytostatic to cytotoxic effects. While compound **13** exhibits broad apoptotic potential across cell lines, compound **12** appears to have a more selective effect on HepG2 cells, suggesting specific interactions with cellular components or regulatory pathways unique to liver cells. In addition, the low necrotic activity across treatments highlights a preference for apoptotic pathways that may minimise the inflammatory responses commonly associated with necrosis. These findings emphasise the therapeutic potential of *ent*-kaurene derivatives, especially compound **13**, as selective apoptotic agents with applicability across diverse cancer cell types. Further investigations into their molecular mechanisms could provide valuable insights into the apoptotic pathways engaged by these compounds. As a result, these *ent*-kaurene derivatives could serve as promising candidates for targeted cancer therapies with apoptosis-focused mechanisms.

#### 2.2.4. Analysis of Cell Cycle

Flow cytometry was used to measure DNA ploidy and examine changes in cell cycle profiles, aiming to confirm the cytostatic effect associated with the inhibitory response of the selected *ent*-kaurenes on cell growth in the three cancer cell lines tested. By analysing the DNA histograms of treated cells stained with propidium iodide (PI), we were able to precisely quantify cell populations across G0/G1, S, and G2/M phases, revealing distinct arrest patterns associated with each compound. The cell cycle effects of *ent*-kaurene derivatives **7**, **12**, and **13** were investigated in B16-F10, HepG2, and HT29 cells after 72 h of treatment at IC_50_ and IC_80_ concentrations. These derivatives, previously identified due to their remarkable antiproliferative activities, were selected based on their efficacy in previous cytotoxicity assays. Cell cycle phase distributions and DNA content analyses were performed to determine whether cell cycle arrest mechanisms contributed to the observed growth inhibition.

**Figure 9:**
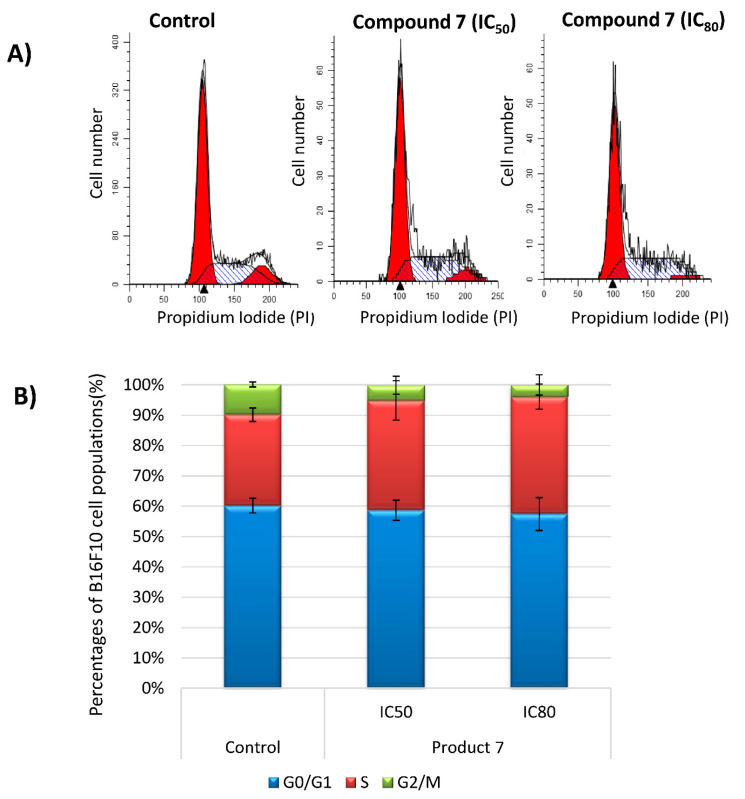
Flow cytometric analysis of the cell cycle of B16F10 melanoma cells after 72h of treatment with compound **7** at IC_50_ and IC_80_. (**A**) Representative histograms obtained via FACS analysis. Red: cells in G0/G1 and G2/M phases; blue diagonal lines: cells in S phase. (**B**) Changes in the percentage of cells in each phase of the cell cycle with respect to the control untreated cells. Values represent the mean ± standard deviation from at least two experiments in triplicate.

The effects of compound **7** on B16-F10 cell cycle distribution were as follows: As shown in Figure 9, compound **7** had a minimal effect on cell cycle phase distribution in B16-F10 cells, with results that were not significant compared to control conditions. Cell populations in the G0/G1, S, and G2/M phases remained similar to those of untreated controls at both IC_50_ and IC_80_ concentrations, suggesting that the cytotoxic effects of compound **7** may occur independently of cell cycle disruption. This observation suggests that there are alternative cytotoxic pathways for compound **7**, possibly through direct mitochondrial disruption or alternative apoptotic pathways not primarily involving cell cycle arrest.

**Figure 10:**
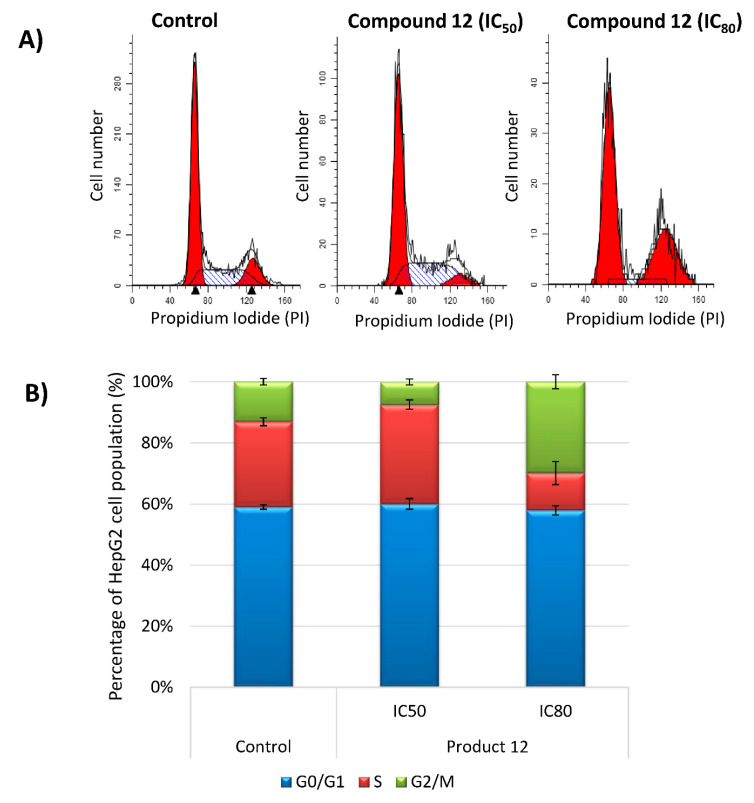
Flow cytometric analysis of the cell cycle of HepG2 hepatocellular carcinoma cells with compound **12** at IC_50_ and IC_80_ concentrations after 72 h of treatment. (**A**) Representative histograms obtained by FACS analysis. Red: cells in G0/G1 and G2/M phases; blue diagonal lines: cells in S phase. (**B**) Changes in the percentage of cells in each phase of the cell cycle with respect to the untreated control cells. Values represent the mean ± standard deviation of at least two experiments in triplicate.

The effects of compound **12** on HepG2 cell cycle arrest and distribution are presented as follows: Treatment with compound **12** resulted in a significant increase in the G2/M phase cell population at IC_80_ concentration, with 17% more cells in G2/M compared to untreated controls, while the S phase population decreased significantly, by approximately 16% (Figure 10). This shift suggests that compound **12** induces a G2/M phase block at higher concentrations, potentially halting DNA synthesis and inhibiting mitosis. However, at IC_50_ concentrations, compound **12** showed limited changes in cell cycle progression, with no significant differences in phase distribution, indicating a concentration-dependent effect on G2/M arrest in HepG2 cells. This result suggests the involvement of the G2/M checkpoint at higher doses, where compound **12** may interfere with the key regulatory proteins required for mitotic entry.

**Figure 11:**
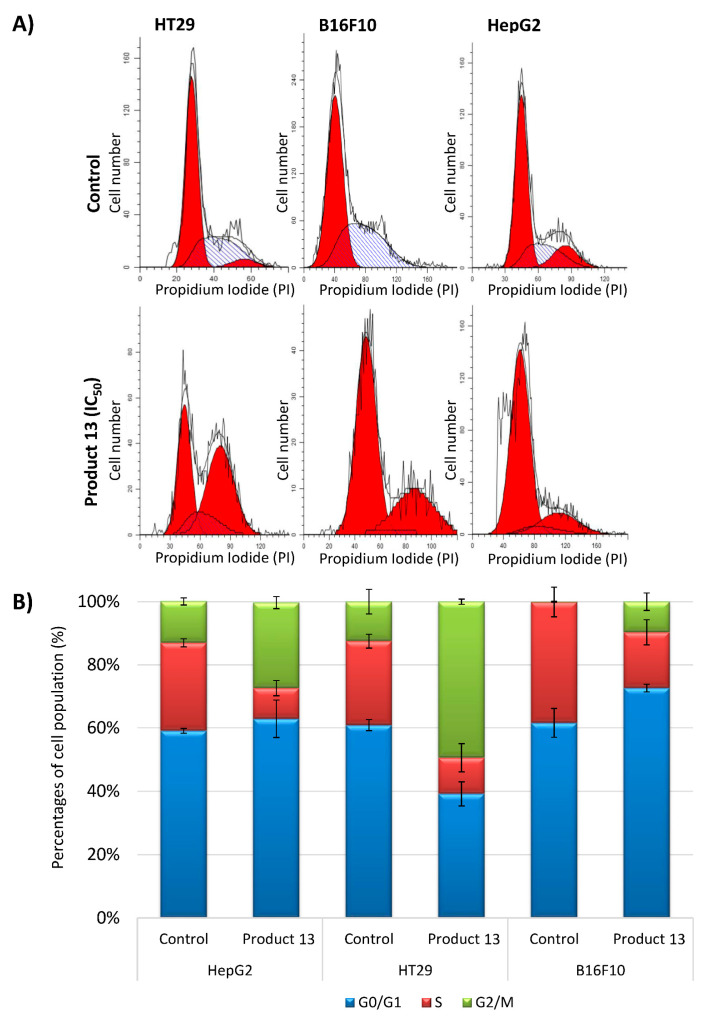
Flow cytometric analysis of the cell cycle of HT29, B16-F10, and HepG2 cancer cells with compound **13** at IC_50_ and IC_80_ concentrations after 72 h of treatment. (**A**) Representative histograms obtained by FACS analysis. Red: cells in G0/G1 and G2/M phases; blue diagonal lines: cells in S phase. (**B**) Changes in the percentage of cells in each phase of the cell cycle with respect to untreated control cells. Values represent the mean ± standard deviation of at least two experiments in triplicate.

The effects of compound **13** on HT29, HepG2, and B16-F10 cell cycle arrest were as follows: Compound **13** showed the strongest effect on cell cycle arrest among the *ent*-kaurenes tested (Figure 11). In HT29 cells, treatment with compound **13** resulted in a significant accumulation in the G2/M phase: approximately 49.4% of the cells were detected in the G2/M phase, an increase of 36.9% compared to control untreated cells. This redistribution was accompanied by a significant reduction in S phase cells of 15% and a 21% reduction in G0/G1 phase cells, indicating an effective blockade at the G2/M checkpoint. In HepG2 cells, compound **13** similarly induced G2/M arrest, increasing the G2/M cell population by 14% while reducing S phase cells by 18%. Interestingly, G0/G1 changes were negligible, suggesting a specific effect on G2/M. This suggests that compound **13** may inhibit critical proteins, such as cyclin-dependent kinases (CDKs).

In B16-F10 cells, compound **13** showed a moderate effect, with G2/M and G0/G1 cell cycle arrest both increasing by approximately 11% each. Correspondingly, S-phase cells decreased by approximtely 21% compared to controls, suggesting a partial G2/M arrest coupled with reduced DNA synthesis. The ability of compound **13** to induce G2/M arrest in all cell lines tested underscores its potential as a universal cytostatic agent, and its consistent effect highlights its selective action on cell cycle regulatory mechanisms. Notably, at the IC_80_ concentration, compound **13** showed no further enhancement of G2/M arrest in any of the cell lines, suggesting that higher doses may favour the activation of apoptotic pathways over cell cycle arrest. This is consistent with the observations of increased apoptosis and markers such as chromatin condensation, membrane blebbing, and apoptotic body formation in parallel microscopy assays, indicating a shift from cytostatic to cytotoxic effects at elevated concentrations.

These cell cycle analyses show that *ent*-kaurene derivatives **12** and **13** exhibit promising cytostatic activity through selective G2/M-phase arrest; this is particularly effective in HT29 and HepG2 cells. The concentration-dependent effects suggest a dual mechanism, whereby the compounds first inhibit cell proliferation and subsequently induce apoptosis at higher doses. Further studies focusing on protein-level interactions, particularly at the G2/M checkpoint, may provide insight into the molecular basis of these responses and provide a basis for the potential therapeutic use of *ent*-kaurenes in targeted cancer treatments.

#### 2.2.5. Analysis of Mitochondrial Membrane Potential

The mitochondrial membrane potential (Δψm) is a critical parameter in assessing mitochondrial function and serves as an early indicator of apoptosis in cells. The disruption of Δψm indicates mitochondrial destabilisation, which is often associated with the intrinsic apoptotic pathway. Therefore, studying changes in mitochondrial membrane potential (MMP) in tumour cells provides valuable insights into the mechanism and pathway of apoptosis induced by anticancer agents. Apoptotic effects can manifest due to the activation of intrinsic (mitochondrial) or extrinsic apoptotic pathways. If apoptosis is accompanied by loss of MMP, this suggests mitochondrial involvement and activation of the intrinsic pathway. Conversely, when apoptosis is induced without changes in MMP, this typically indicates activation by the extrinsic pathway, often mediated by cell-surface-death receptors.

**Figure 12:**
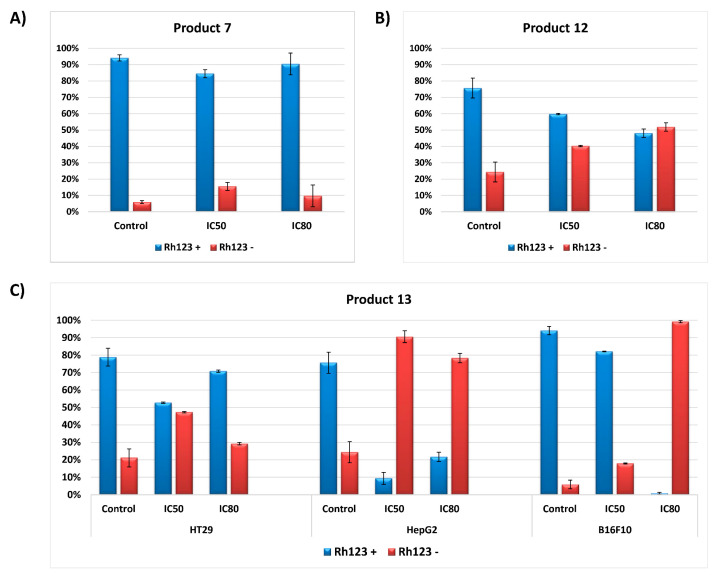
Effects of products **7**, **12**, and **13** on the mitochondrial membrane potential. Compound **7** on B16-F10 (**A**), compound **12** on HepG2 (**B**), and compound **13** on HT29, HepG2, and B16-F10 cancer cell lines (**C**). The experiment was performed at IC_50_ and IC_80_ concentrations after 72 h treatment. The percentage of Rh123-positive or -negative cells relative to untreated control cells (control) is shown. Values are expressed as mean ± SD of two independent experiments performed in triplicate.

In this study, MMP changes were analysed in HT29, HepG2, and B16-F10 cell lines after treatment with compounds **7**, **12**, and **13**. Flow cytometry with Rhodamine 123 (Rh123) and propidium iodide (PI) double-staining was used to assess MMP integrity. Rhodamine 123 is a lipophilic cationic dye that selectively accumulates in mitochondria, and the logarithmic ratio of the probe concentration between the inner and outer mitochondria membrane is proportional to the mitochondrial membrane potential. A decrease in Rh123 fluorescence indicates a loss of mitochondrial potential, signalling mitochondrial dysfunction. After 72 h exposure to compounds **7**, **12**, and **13** at IC_50_ and IC_80_ concentrations, the percentage of Rh123-positive and Rh123-negative cells was determined, providing a comparative measure of MMP integrity relative to untreated controls (Figure 12).

The effects of compound **7** on B16-F10 cells were as follows: The MMP analysis for compound **7** on B16-F10 melanoma cells showed no significant reduction in Rh123 fluorescence. Both IC_50_ and IC_80_ concentrations produced high Rh123-positive staining, consistent with untreated cells. This lack of MMP disruption implies that compound **7** does not induce mitochondrial destabilisation in B16-F10 cells, suggesting that apoptosis is likely to be mediated by an extrinsic apoptotic pathway, independent of mitochondrial influence. This extrinsic pathway activation could involve cell-surface-death receptors such as Fas ligand or tumour necrosis factor receptor (TNF-R), bypassing the mitochondrial apoptotic cascade.

The effects of compound **12** on HepG2 cells were as follows: In HepG2 cells, compound **12** induced a significant decrease in Rh123-positive cell populations. The increase in Rh123-negative cells at both IC_50_ and IC_80_ concentrations suggests significant MMP loss, suggesting that compound **12** induces apoptosis via the intrinsic or mitochondrial pathway in hepatocarcinoma cells. The dose-dependent increase in mitochondrial dysfunction may reflect early mitochondrial membrane permeability changes, cytochrome c release, and the activation of downstream caspases, all hallmarks of intrinsic apoptosis. This mitochondria-targeted mechanism is consistent with the broader apoptotic efficacy of compound **12** in HepG2 cells.

The effects of compound **13** in all cell lines were as follows: Compound **13** demonstrated the most potent effect on mitochondrial membrane potential among all compounds tested in all three cell lines. In HT29 colon adenocarcinoma cells, treatment with compound **13** resulted in a significant reduction in Rh123-positive cells at both IC_50_ and IC_80_ concentrations, indicating a pronounced loss of MMP and suggesting intrinsic apoptosis. These results correlate with the high apoptosis rates observed in HT29 cells, suggesting that compound **13** exerts a robust pro-apoptotic effect through mitochondrial destabilisation. In HepG2 cells, compound **13** also induced a dose-dependent loss of MMP, as evidenced by the significant increase in the Rh123-negative population compared to untreated controls. This decrease in MMP at both concentrations highlights the potent mitochondrial action of compound **13**, which may involve mitochondrial membrane permeabilisation and the release of pro-apoptotic factors. In contrast, in B16-F10 melanoma cells, compound **13** induced a marked increase in the Rh123-negative population at the IC_80_ concentration, indicating MMP destabilisation and the activation of intrinsic apoptosis, although this effect was not observed at the IC_50_ concentration, which could mean that, in this cell line, there may be an initial activation of the extrinsic apoptosis-inducing extrinsic pathway and a secondary activation of the intrinsic pathway. Future studies are required to clarify this point. These consistent effects across cell lines support the role of compound **13** as a broad-spectrum mitochondrial disruptor.

Comparative analysis of the MMP effects between compounds shows that compounds 5 and 7 induce apoptosis primarily through the mitochondrial pathways in HepG2 and HT29 cells, respectively, with compound **13** exerting this effect in all cell lines tested. In contrast, the lack of effect of compound **7** on MMP in B16-F10 cells suggests an alternative apoptotic pathway, probably extrinsic in nature. These findings suggest compound-specific mechanisms of action, with compounds **12** and **13** acting as potent inducers of mitochondrial destabilisation, whereas compound **7** bypasses mitochondrial involvement. The consistent induction of MMP degradation via compounds **12** and **13** highlights their potential as selective mitochondrial targeting agents for cancer therapy. Future investigations could focus on elucidating the specific molecular targets within the mitochondrial apoptotic pathway, including the role of Bcl-2 family proteins, caspase activation and cytochrome c release. The differential effects observed between the compounds suggest the utility of *ent*-kaurene derivatives in tailored cancer therapies that exploit apoptotic pathways to selectively induce apoptosis in cancer cells while minimising non-specific toxicity.

## 3. Discussion

The significant anticancer potential of *ent*-kaurene diterpenoids, particularly compounds **7**, **12**, and **13**, has been demonstrated in the assayed three cancer cell lines: colorectal (HT29), liver (HepG2), and melanoma (B16-F10) cancer cell lines. Also, we analysed the anticancer activity and mechanisms of action of compounds isolated and structurally modified from *D. tenuifolium*, supporting their potential as promising therapeutic agents for a range of cancers.

Cell viability assays revealed a range of cytotoxic potentials for the investigated compounds, with IC_50_ values ranging from 2.12 μM to 153.83 μM. Among these, compound **13** emerged as the most potent cytotoxic agent, demonstrating a dose-dependent anti-proliferative effect in all cancer cell lines tested (HT29, HepG2, and B16-F10). In HT29 cells, compound **13** achieved an impressive IC_50_ of 2.71 ± 0.23 µM, significantly outperforming compounds **2** and **12**, which exhibited IC_50_ values of 10.08 ± 2.69 µM and 45.37 ± 5.34 µM, respectively. Notably, compound **12** exhibited selective cytotoxicity towards HepG2 cells, suggesting its potential application in the treatment of hepatocellular carcinoma. This observation is consistent with previous findings on other diterpenoids, such as kaurenoic acid, which demonstrated cell line-specific activity influenced by structural modifications [21]. Structurally, this compound is characterised by the presence of an exo-methylene cyclopentanone, which is consistent with previous studies highlighting the critical role of this moiety in the cytotoxic activity of *ent*-kaurene diterpenoids [27].

Oridonin, one of the most potent tetracyclic *ent*-kaurenes, was isolated from the genus Isodon and one of its derivatives has progressed to phase I clinical trials [28]. Oridonin shows activity against several types of cancer, including acute myeloid leukaemia, pancreatic, oesophageal, nasopharyngeal, colorectal, and liver cancer, with an IC_50_ of 25.7 µM in HepG2 cells [29]. Another example is kaurenic acid, isolated from Espeletia semiglobulata, which showed significant antimelanoma activity, with an IC_50_ of 0.79 µM in B16-F10 cells. In addition, ponicidin, extracted from Isodon adenolomus, was identified as a cytotoxic agent against hepatocellular carcinoma, lung cancer, monocytic leukaemia, and colorectal cancer. Treatment with ponicidin was reported to significantly suppress the growth of HT29 cells (~4-fold reduction), highlighting its potential as an anticancer agent [30,31,32].

A cell cycle analysis showed that compounds **12** and **13** induced a marked G2/M cell cycle arrest in HT29 and HepG2 cell lines in a dose-dependent manner, highlighting their cytostatic potential. G2/M arrest is a well-documented mechanism for stopping cancer cell proliferation through preventing mitotic entry and allowing for DNA damage repair or apoptotic signalling. In HT29 cells, compound **13** caused a significant 49.4% increase in the G2/M population, accompanied by a reduction in the S and G0/G1 phases. Similarly, compound **12** induced a 17% increase in the G2/M population in HepG2 cells at its IC_80_ concentration, further emphasising its role in arresting mitotic progression. These results suggest that *ent*-kaurenoids interfere with mitotic entry by modulating cyclin-dependent kinases (CDKs) and checkpoint regulators [33]. Interestingly, the G2/M arrest observed with these compounds is similar to the effects of oridonin, a structurally related diterpenoid known to inhibit microtubule dynamics and spindle assembly checkpoints [34]. This common mechanism of action among diterpenoids underscores their potential as inhibitors of mitotic progression and highlights their therapeutic relevance in cancer treatment [20].

Our findings are consistent with previous research on pharicin A, a novel natural *ent*-kaurenoid isolated from *Isodon pharicus*, which has been identified as a cytostatic and cytotoxic agent. Pharicin A induces G2/M phase arrest in both paclitaxel-sensitive and resistant tumour cells, an effect attributed to its ability to bind BubR1, disrupt its subcellular localisation, and inhibit its kinase activity [20]. Similarly, other studies have shown that *ent*-kaurenoids can induce cell cycle arrest and apoptosis while exhibiting moderate to low toxicity in normal cell lines. [35] Many cytotoxic compounds exert inhibitory effects on cancer cell growth by arresting the cell cycle at specific checkpoints, inducing apoptosis, or a combination of both mechanisms [26,36,37].

One of the most compelling findings of this study was the strong apoptotic induction observed with compounds **7**, **12**, and **13**, particularly compound **13**, which exhibited broad apoptosis rates across multiple cell lines, with total apoptosis percentages ranging from 20% to 99%. In HepG2 cells, compound **13** achieved total apoptosis rates of over 95%, with a dose-dependent progression from the early to late apoptotic stages. Apoptosis studies using Annexin V-FITC/PI staining and flow cytometry confirmed these results and demonstrated that the apoptotic effects of compounds **12** and **13** were strongly associated with mitochondrial dysfunction, as evidenced by the loss of Δψm in treated cells. The reduction in Rh123 fluorescence in HT29 and HepG2 cells further supports the activation of the intrinsic apoptotic pathway, highlighting the mechanistic involvement of mitochondrial disruption in their cytotoxic effects.

A comparative analysis of mitochondrial membrane potential (MMP) effects revealed that compounds **12** and **13** induced apoptosis primarily through mitochondrial pathways in HepG2 and HT29 cells, respectively, with compound **13** exerting this effect in all cell lines tested. Mitochondrial destabilisation, as evidenced by a significant decrease in Rh123 fluorescence, was particularly pronounced in HT29 and HepG2 cells. This effect is likely to correlate with the release of pro-apoptotic factors such as cytochrome c into the cytosol, leading to caspase activation.

In contrast, compound **7** induced apoptosis in B16-F10 cells without significant MMP disruption, suggesting an alternative, extrinsic apoptotic pathway, possibly mediated by death receptors such as Fas or TNFR, which operate independently of mitochondrial involvement. These results highlight the compound specific mechanisms of action, with compounds **12** and **13** acting as potent inducers of mitochondrial destabilisation, whereas compound **7** appears to bypass the mitochondrial pathways. Notably, compound **13**, which exhibited the strongest apoptotic effect, contains an α,β-unsaturated ketone group, a structural feature that may explain its efficacy in all tumour cell lines tested. Further molecular studies will be essential to confirm these mechanistic findings and to fully elucidate the apoptotic pathways involved.

Consistent with these findings, previous studies on the apoptotic effects of *ent*-kaurenoids suggest that these biomolecules primarily activate intrinsic apoptotic mechanisms, particularly in compounds containing an α,β-unsaturated ketone group. Oridonin and related compounds have been shown to induce apoptosis through a mitochondrial-related pathway, in which cytochrome c plays a central role. The α,β-unsaturated ketone moiety was identified as an active functional group that is critical for their cytotoxicity, mitochondrial localisation, and uptake [38]. Furthermore, there is evidence supporting mitochondrial redox changes as a potential mediator of the apoptotic activities of oridonin in HepG2 cells [39].

From a chemical perspective, the widely accepted mechanism of action of these diterpenoids involves the Michael addition of soft nucleophiles, such as thiols and protein sulfhydryl (SH) groups, to the α,β-unsaturated ketone moiety. This reaction deactivates SH-dependent enzymes and coenzymes, leading to the accumulation of reactive oxygen species (ROS). The deactivation of SH enzymes and coenzymes disrupts intracellular redox homeostasis, while elevated ROS levels induce apoptosis through oxidative damage to tumour cells [40]. The importance of the α,β-unsaturated ketone group was highlighted in the potent induction of mitochondrial apoptosis and promotion of ROS generation in cancer cells [29]. For example, pteisolic acid G, a novel *ent*-kaurene diterpenoid, was reported to inhibit cell viability and induce apoptosis in human colorectal cancer cells through an intrinsic apoptotic mechanism. Polyisolic acid G downregulated anti-apoptotic proteins such as Bcl-2 and Bcl-XL and factors such as NF-κB p65 and p-p65. Conversely, it upregulated pro-apoptotic proteins, including Puma, Bax, and Bim, and the tumour suppressor p53.

These differential effects highlight the versatility of *ent*-kaurene derivatives in targeting multiple apoptotic pathways. The ability of *ent*-kaurene derivatives to target both intrinsic and extrinsic pathways increases their therapeutic versatility and may overcome the resistance mechanisms in apoptosis-deficient cancers. A similar dual-pathway activation was observed in other diterpenoids, such as triptolide, which simultaneously activates mitochondrial dysfunction and death receptor signalling [41]. This highlights the potential of *ent*-kaurene derivatives as multi-target agents capable of addressing the heterogeneity of cancer. Future investigations could focus on elucidating the specific molecular targets within the mitochondrial apoptotic pathway, including the role of Bcl-2 family proteins, caspase activation, and cytochrome c release. The differential effects observed between compounds **7**, **12**, and **13** suggest the utility of *ent*-kaurene derivatives in tailored cancer therapies that exploit apoptotic pathways to selectively induce apoptosis in cancer cells while minimising non-specific toxicity.

## 4. Materials and Methods

### 4.1. Chemistry

#### 4.1.1. General Experimental Procedures

Thin-layer chromatography (TLC) was performed using F254 precoated plates (0.25 mm) and visualised using UV fluorescence quenching and phosphomolybdic acid solution in ethanol staining. Flash chromatography was performed on silica gel (230–400 mesh). Chromatography separations were carried out via conventional column on silica gel 60 (230–400 Mesh), using hexanes-AcOEt (AcOEt-hexane) mixtures of increasing polarity. ^1^H and ^13^C NMR spectra were recorded on a Bruker Nanobay Avance NEO III HD High Definition NMR Spectrometer, Billerica, MA, USA, at 500 MHz and at 125 MHz, respectively. CDCl_3_ was treated with K_2_CO_3_. Chemical shifts (δ H) are quoted in parts per million (ppm), noting the appropriate residual solvent peak and tetramethylsilane. Data for ^1^H NMR spectra are reported with the abbreviations s, br s, d, br d, t, dt, dq, and m denoting singlet, broad singlet, doublet, broad doublet, triplet, doublet triplet, doublet quartet, and multiplet, respectively. *J* = coupling constant in Hertz (Hz). Data for ^13^C NMR spectra are reported in terms of chemical shift relative to Me_4_Si (δ 0.0) and the signals were assigned utilising DEPT experiments and on the basis of heteronuclear correlations. Infrared spectra (IR) were recorded as thin films or as solids on a Perkin Elmer model, USA. One FTIR spectrophotometer with samples between sodium chloride plates or as potassium bromide pellets and are reported in terms of their frequency of absorption (cm^−1^). Only selected absorbances (ν_max_) are reported.

#### 4.1.2. Extraction and Isolation

The plant was collected in the north of Granada, Spain. The air-dried aerial parts of *D. tenuifolium* (158.25 g) was powdered and extracted with 500 mL of MeOH at room temperature. The MeOH extract was evaporated under reduced pressure to yield a crude extract of 43.1g. The extract was subjected to column chromatography on silica gel, eluting with an increasingly polar gradient of ethyl acetate (AcOEt) to yield 10.1 g of **7** (10% AcOEt/hexane) and 250 mg of **8** (15% AcOEt/hexane).

#### 4.1.3. Synthesis and Structural Characterisation

##### Structural Characterisation of Compounds (**7**) and (**8**)

*ent*-15α-angeloyloxykaur-16-en-3β-ol (**7**): white solid; ^1^H NMR data (CDCl_3_, 500 MHz) (see Table S1, included in the Supplementary Materials); ^13^C NMR data (CDCl_3_, 125 MHz) (see Table S2, included in the Supplementary Materials). HRMS (ESI) *m*/*z* calcd for C_25_H_38_O_3_Na (M+Na^+^) 409.2719 was 409.2727. The spectroscopic data of **7** were obtained using those described in the literature [23].

*ent*-15*α*-angeloyloxykaur-16-en-3*β*,9*α*-diol (**8**): white solid; ^1^H NMR data (acetone-*d*_6_, 500 MHz) (see Table S1, included in the Supplementary Materials); ^13^C NMR data (acetone-*d*_6_, 125 MHz) (see Table S2, included in the Supplementary Materials). HRMS (ESI) *m/z* calcd for C_25_H_38_O_4_Na (M + Na^+^) 425.2668 was 425.2680. Spectroscopic data of **8** were obtained according to the description in the literature [7].

##### Synthesis of Acetate **9**

Ac_2_O (0.5 mL) was added to a solution of alcohol 7 (123 mg, 0.318 mmol) in pyridine (1 mL) and the mixture was stirred for 2 h. Then, water (5 mL) was added, and the mixture was stirred for 5 min. Next, it was diluted with AcOEt (15 mL), and the phases were shaken and separated. The organic phases were washed with 2 N HCl (3 × 5 mL), saturated NaHCO_3_ (3 × 5 mL), and brine (5 mL), and dried over anhydrous Na_2_SO_4_. Removal of the solvent under vacuum yielded a crude product (138 mg). Flash chromatography (20% AcOEt/hexane) yielded **9** (130 mg, 96%) as a colourless syrup. ^1^H NMR (CDCl_3_, 400 MHz); δ 0.78 (dd, *J* = 11.5, 1.6 Hz, 1H); 0.86 (s, 6H); 1.02 (m, 1H); 1.09 (s, 3H); 1.20 (m, 1H); 1.92 (dt, *J*= 13.3, 3.2, 3.2 Hz, 1H); 1.95 (br s, 3H); 2.06 (q, *J* = 7.2 Hz, 3H); 2.06 (s, 3H); 1.22–1.81 (m, 12H); 2.71 (br s, 1H); 4.48 (dd, *J* = 11.0, 5.0 Hz, 1H); 4.92 (br s, 1H); 4.95 (br s, 1H); 5.27 (t, *J*= 2.5 Hz, 1H); 6.14 (br q, *J* = 7.2 Hz, 1H). ^13^C NMR (125 MHz, CDCl_3_): 15.9 (CH_3_); 16.6 (CH_3_); 17.7 (CH_3_); 17.8 (CH_2_); 19.4 (CH_2_); 20.8 (CH_3_); 21.3 (CH_3_); 23.6 (CH_2_); 28.3 (CH_3_); 33.4 (CH_2_); 36.5 (CH_2_); 37.7 (C); 38.6 (CH_2_); 38.6 (CH_2_); 38.6 (C); 40.7 (CH); 46.0 (C); 48.1 (CH); 54.9 (CH); 80.9 (CH); 81.1 (CH); 106.3 (CH_2_); 127.9 (C); 138.4 (CH); 153.7 (C); 168.1 (C); 171.0 (C). HRMS (ESI) *m*/*z* calcd for C_27_H_40_O_4_Na (M + Na^+^) 451.2824 was 451.2823.

##### Synthesis of Ketone **10**

*Jones* reagent (0.2 mL) was added dropwise at 0 °C to a solution of compound 7 (187 mg, 0.48 mmol) in acetone (4 mL) and the reaction mixture was stirred for 10 min, at which point no starting material for TLC was found. The solvent was then removed under vacuum, and the crude product was diluted with AcOEt–water (20:10 mL). The phases were shaken and separated, and the organic layer was washed with water (3 × 10 mL) and brine and dried over anhydrous Na_2_SO_4_. Removal of the solvent under vacuum provided a crude product that was purified using flash chromatography to yield **10** (173 mg, 93%) as a colourless syrup.^1^H NMR (CDCl_3_ 400 MHz); δ 1.04 (s, 3H); 1.08 (s, 3H); 1.13 (s, 3H); 1.94 (br s, 3H); 2.05 (q, *J* = 7.2 Hz, 3H); 2.06 (s, 3H); 1.18–2.19 (m, 12H); 2.45–2.53 (m, 2H); 2.74 (br s, 1H); 4.94 (br s, 1H); 4.97 (br s, 1H); 5.31 (t, *J*= 2.5 Hz, 1H); 6.13 (dq, *J* = 7.2, 1.4 Hz, 1H). ^13^C NMR (125 MHz, CDCl_3_): 15.8 (CH_3_); 17.8 (CH_3_); 18.2 (CH_2_); 20.8 (CH_3_); 21.0 (CH_3_); 21.1 (CH_2_); 27.2 (CH_3_); 33.2 (CH_2_); 34.1 (CH_2_); 36.2 (CH_2_); 37.8 (C); 38.2 (CH_2_); 39.5 (CH_2_); 40.6 (CH); 45.9 (C); 47.1 (C); 54.1 (CH); 81.0 (CH); 106.5 (CH_2_); 127.8 (C); 138.5 (CH); 153.5 (C); 168.0 (C); 217.8 (C). HRMS (ESI) *m/z* calcd for C_25_H_36_O_3_Na (M + Na^+^) 407.2562 was 407.2572.

##### Synthesis of Diketone **11**

An O_3_/O_2_ mixture was slowly bubbled through a stirred solution of **10** (113 mg, 0.294 mmol) in dichloromethane (10 mL) at −78 °C for 20 min. At this point, no remaining TLC starting material was found. Then, the excess ozone was removed by bubbling the solution with argon, PPh_3_ (105 mg, 0.4 mmol) was added at −78 °C, the reaction mixture was stirred for 2 h at room temperature, and the solvent was evaporated under vacuum to obtain a crude product. Purification via chromatography on silica gel (20% AcOEt/hexane) yielded pure diketone **11** (59 mg, 52%) as a colourless syrup. ${\left[\alpha \right]}_{D}^{25}$ = -28.3 (c 0.38, CHCl_3_). IR (film) ʋ_max_: 2929, 1759, 1703, 1448, 1373, 1136, 1111, 1025, 978 cm^−1^. ^1^H NMR (CDCl_3_, 400 MHz); δ 1.07 (s, 3H); 1.10 (s, 3H); 1.19 (s, 3H); 1.27 (br s, 3H); 1.49 (q, *J* = 7.2 Hz, 3H); 1.90 (m, 1H); 2.09 (m, 1H); 2.40 (m, 1H); 2.47–2.55 (m, 2H); 2.58 (br s, 1H); 4.91 (s, 1H); 5.40 (q, *J* = 7.2 Hz; 1H). ^13^C NMR (125 MHz, CDCl_3_): 15.5 (CH_3_); 18.1 (CH_3_); 18.8 (CH_3_); 18.9 (CH_2_); 20.0 (CH_2_); 21.1 (CH_3_); 27.2 (CH_3_); 29.8 (CH_2_); 32.6 (CH_2_); 33.9 (CH_2_); 37.8 (CH_2_); 38.3 (C); 39.2 (CH_2_); 44.0 (CH); 45.0 (C); 46.1 (CH); 47.1 (C); 54.1 (CH); 78.8 (CH); 82.6 (CH); 102.5 (CH); 104.6 (C); 168.2 (C); 213.9 (C); 217.1 (C). HRMS (ESI) *m/z* calcd for C_24_H_34_O_4_Na (M + Na^+^) 409.2355 was 409.2363.

##### Synthesis of Hydroxyketone **12**

An O_3_/O_2_ mixture was slowly bubbled through a stirred solution of **7** (87 mg, 0.225 mmol) in dichloromethane (20 mL) at −78 °C for 30 min. At this point, no remaining TLC starting material was observed. The excess ozone was then removed via bubbling the solution with argon, and PPh_3_ (393 mg, 1.5 mmol) was added at −78 °C. Next, the reaction mixture was stirred for 4 h at room temperature. Hydroxyketone **12** (34 mg, 39%) was obtained following the same method as that used for 11 after chromatography, using (30% AcOEt/hexane) as the eluent. ${\left[\alpha \right]}_{D}^{25}$ −46.0 (c 0.05, CHCl_3_).IR (film) ʋ_max_: 3492, 2932, 1759, 1453, 1276, 1139, 981, 680 cm^−1^. ^1^H NMR (CDCl_3_, 400 MHz); δ 0.81 (s, 3H); 0.99 (s, 3H); 1.11 (s, 3H); 1.25 (br s, 3H); 1.49 (q, *J* = 7.2 Hz, 3H); 1.36–2.02 (m, 15H); 2.53 (br s, 1H); 3.21 (m, 1H); 4.89 (br s, 1H); 5.40 (br q, *J* = 7.2 Hz, 1H). ^13^C NMR (125 MHz, CDCl_3_): 15.5 (CH_3_); 15.6 (CH_3_); 18.0 (CH_3_); 18.3 (CH_2_); 18.6 (CH_2_); 18.9 (CH_3_); 27.1 (CH_2_); 28.4 (CH_3_); 30.0 (CH_2_); 32.9 (CH_2_); 38.7 (CH_2_); 38.8 (CH_2_); 38.8 (CH_2_); 38.9 (C); 44.1 (CH); 45.1 (C); 47.1 (CH); 54.7 (CH); 78.8 (CH); 82.8 (CH); 102.5 (CH_2_); 104.6 (C); 168.3 (CH); 2014.3 (C). HRMS (ESI) *m/z* calcd for C_24_H_36_O_4_Na (M + Na^+^) 411.2511 was 411.2512.

##### Synthesis of Diol **2**

LiAlH_4_ (190 mg, 5.05 mmol) was carefully added to a solution of **7** (484 mg, 1.25 mmol) in anhydrous THF (10 mL), previously kept cold at 0 °C, and the reaction mixture was stirred at room temperature for 90 min. At this point, no remaining TLC starting material was observed. The mixture was poured onto ice and washed with AcOEt (3 × 20 mL). The mixture was transferred to a separating funnel and the phases were shaken and separated. The aqueous phase was extracted using AcOEt (10 mL) and the organic phases were combined. Then, it was washed with water and brine and dried over anhydrous Na_2_SO_4_. The removal of the solvent under vacuum yielded a crude product that was chromatographed on silica gel (30% AcOEt/hexane) to yield pure diol **2** (346 mg, 91%) as a white amorphous solid. ^1^H NMR (CDCl_3_, 400 MHz) *δ* 0.79 (s, 3H); 1.00 (s, 3H); 1.05 (s, 3H); 1.06 (ddd, *J*= 13.1, 13.1, 4.5 Hz, 1H); 1.28–1.95 (m, 15H); 1.99 (d, *J* = 15.0 Hz, 1H); 2.67 (br s, 1H); 3.23 (dd, *J* = 11.2, 5.1 Hz, 1H); 3.77 (s, 1H); 4.98 (s, 1H), 1H); 5.11 (s, 1H); ^13^C NMR (CDCl_3_, 125 MHz) *δ* 15.5 (CH_3_); 17.6 (CH_3_); 18.2 (CH_2_); 19.7 (CH_2_); 27.3 (CH_2_); 28.3 (CH_3_); 33.2 (CH_2_); 36.4 (CH_2_); 38.6 (CH_2_); 38.7 (CH_2_); 38.8 (C); 38.8 (C); 40.1 (CH); 45.6 (C); 46.3 (CH); 54.5 (CH); 79.0 (CH); 82.3 (CH); 104.9 (CH_2_); 158.3 (C). HRMS (ESI) *m/z* calcd for C_20_H_32_O_2_Na (M + Na^+^) 327.2300 was 327.2308.

##### Synthesis of Hydroxyenone **13**

MnO_2_ (1 g, 11.49 mmol) was added to a solution of diol **2** (267 mg, 0.88 mmol) in CHCl_3_ (10 mL), and the mixture was stirred at reflux for 1 h. The filtrate was then filtered through silica gel (5 g), washed with AcOEt (10 mL), and concentrated to yield hydroxyenone **13** (246 mg, 93%) as a white solid. ^1^H NMR (CDCl_3_ 400 MHz); δ 0.78 (s, 3H); 1.00 (s, 3H); 1.08 (s, 3H); 0.82–1.97 (m, 17H); 2.39 (d, *J*= 11.9 Hz, 1H, 3H); 3.04 (br s, 1H); 3.20 (dd, *J*= 11.3, 5.0 Hz, 1H); 5.25 (s, 1H); 5.94 (s, 1H). ^13^C NMR (125 MHz, CDCl_3_): 15.4 (CH_3_); 17.5 (CH_3_); 18.2 (CH_2_); 18.4 (CH_2_); 27.0 (CH_2_); 28.2 (CH_3_); 32.3 (CH_2_); 33.5 (CH_2_); 36.6 (CH_2_); 38.0 (CH); 38.1 (CH_2_); 38.8 (C); 39.7 (C); 52.2 (C); 52.5 (CH); 54.2 (CH); 78.7 (CH); 114.6 (CH_2_); 149.4(C); 210.9 (C). HRMS (ESI) *m*/*z* calcd for C_20_H_30_O_2_Na (M + Na^+^) 325.2143 was 325.2140.

### 4.2. Biological Experimental Procedures

#### 4.2.1. Materials Used

Dulbecco’s modified Eagle’s medium (DMEM), RPMI 1640 medium with L-glutamine, foetal bovine serum (FBS), penicillin/streptomycin (Biowest, Nuaillé, France), dimethylsulfoxide (DMSO, Merck Life Science S.L., Madrid, Spain), and 3-(4,5-dimethylthiazol-2-yl)-2,5-diphenyltetrazolium bromide (MTT) were purchased from Thermo Fisher Scientific Inc. (Ward Hill, MA, USA). Cell culture flasks and multiwell plates were purchased from VWR International Ltd. (Radnor, PA, USA). All reagents were of analytical grade.

#### 4.2.2. Test Compounds

The test compounds (**2**, **7**–**9**, **11**–**13**) were initially dissolved in DMSO (25% in PBS). Stock solutions were stored at −20 °C until use and diluted in cell culture medium to achieve the appropriate working concentrations for each experimental assay. For the antiproliferative assay performed on all cell lines, the concentrations of the compounds required to achieve a 20%, 50%, and 80% inhibition of cell growth (IC_20_, IC_50_, and IC_80_, respectively) were determined. This approach was designed to evaluate the full range of cytotoxicity and to assess graded versus acute cellular responses to these compounds. Untreated control cells were included in all experiments for comparative analysis. All assays were performed in triplicate to ensure reproducibility and data are expressed as mean ± standard deviation.

#### 4.2.3. Cell Culture and Viability Assay

The human colorectal adenocarcinoma cell line HT29 (ECACC no. 9172201; ATCC no. HTB-38), the human hepatocarcinoma cell line HepG2 (ECACC no. 85011430), and the mouse melanoma cell line B16-F10 (ATCC no. CRL-6475) were cultured in Dulbecco’s modified Eagle’s medium (DMEM), supplemented with 2 mM L-glutamine, 10% heat-inactivated foetal bovine serum (FBS), and antibiotics (10,000 units/mL penicillin and 10 mg/mL streptomycin). Cells were maintained at 37 °C in a humidified atmosphere of 5% CO_2_. Media were changed every 48 h and confluent cultures were subcultured with 0.25% trypsin–EDTA solution. For all experiments, cells were grown as monolayers to 80-90% confluence in sterile cell culture flasks. All cell lines were obtained from the cell bank of the University of Granada, Spain, and authenticated to confirm their identity and mycoplasma-free status.

Cytotoxicity was assessed using the MTT (3-(4,5-dimethylthiazol-2-yl)-2,5-diphenyltetrazolium bromide) assay. Fresh cell suspensions were prepared and seeded into 96-well plates at a density of 6.0 × 10^3^ cells/well for HT29, 5.0 × 10^3^ cells/well for B16-F10, and 1.5 × 10^4^ cells/well for HepG2, with a final volume of 100 µL per well. Cell attachment was achieved via incubation for 24 h at 37 °C in a humidified atmosphere containing 5% CO_2_. Test compounds were prepared in fresh growth medium. After 24 h, 100 µL of medium containing the test compounds at different final concentrations (0–100 µg/mL) were added to the corresponding wells, while control wells received only medium. Cells were incubated with the compounds for 72 h under the same conditions. After incubation, the medium was removed and 100 µL of MTT solution (0.5 mg/mL in a 1:1 mixture of PBS and medium) was added to each well. The plates were further incubated for 1.5 h at 37 °C and the resulting formazan crystals were solubilized by adding 100 µL DMSO per well.

Cell viability relative to untreated controls was quantified by measuring absorbance at 570 nm using an ELISA plate reader (Tecan Sunrise MR20-301, TECAN, Grödig, Austria). Each experiment was performed in triplicate and data were expressed as mean ± standard deviation. Compounds with low IC_50_ values (compounds **1**, **5**, and **7**) were selected for further flow cytometric assays, including apoptosis analysis, cell cycle distribution, and mitochondrial membrane potential determination.

#### 4.2.4. Cell Cycle Analysis

The method used to quantify the amount of DNA in the different phases of the cell cycle (G0/G1, S, and G2/M) was flow cytometry after propidium iodide (PI) staining. For this assay, cells were seeded and allowed to adhere for 24 h prior to treatment. After 24 h, cells were treated with IC_50_ and IC_80_ concentrations of compounds (1, 5, and 7) for 72 h. The cells were then washed twice with PBS, trypsinised and resuspended in 1 × TBS (10 mM Tris and 150 mM NaCl). A Vindelov buffer (100 mM Tris, 100 mM NaCl, 10 mg/mL RNase and 1 mg/mL PI, at pH 8) was then added. Cells were stored on ice and samples were stained with 20 µL of 1 mg/mL PI solution immediately before measurement. Approximately 10 × 10³ cells per sample were analysed in each experiment. Experiments were performed in triplicate, with two replicates per assay. The number of cells at each stage of the cell cycle was estimated using fluorescence-associated cell sorting (FACS) at 488 nm in an Epics XL flow cytometer (Coulter Corporation, Hialeah, FL, USA).

#### 4.2.5. Apoptosis Analysis

To confirm the pro-apoptotic effect of compounds (**1**, **5** and **7**), annexin V-FITC and propidium iodide (PI) double staining was detected via flow cytometry. An early indicator of apoptosis is the translocation of the membrane phospholipid phosphatidylserine from the cytoplasmic interface to the outer surface of the plasma membrane. This event can be specifically detected using annexin V, which binds to phosphatidylserine exposed on the outer leaflet of the plasma membrane. The phospholipid phosphatidylserine that accumulates on the outer plasmatic membrane can be detected by Annexin V/PI, where PI is a fluorescent dye that binds to the nuclei of dead cells. Apoptosis was assessed via flow cytometry using a FACScan (fluorescence-activated cell sorter) flow cytometer (Coulter Corporation, Hialeah, FL, USA).

For this assay, approximately 5 × 10^4^ B16-F10, HT29 and HepG2 cells were plated in 24-well plates with 1.5 mL of medium and incubated for 24 h. Cells were then treated with the selected compounds in triplicate for 72 h at their respective IC_50_ and IC_80_ concentrations. Cells were collected and resuspended in binding buffer (10 mM HEPES/NaOH, pH 7.4, 140 mM NaCl, 2.5 mM CaCl_2_). Annexin V-FITC conjugate (1 µg/mL) was then added and incubated for 15 min at room temperature in the dark. Immediately before flow cytometric analysis, cells were stained with 5 µL of 1 mg/mL PI solution. Approximately 10 × 10³ cells were analysed in each experiment, and the experiment was replicated in triplicate.

#### 4.2.6. Flow Cytometric Analysis of the Mitochondrial Membrane Potential

The electrochemical gradient across the mitochondrial membrane was studied via analytical flow cytometry using the fluorescent probe dihydrorhodamine 123 (DHR 123). DHR is oxidised upon contact with the mitochondria of living cells to form a highly fluorescent product called rhodamine 123 (Rh123). The emitted fluorescence can be monitored via fluorescence spectroscopy using excitation and emission wavelengths of 500 and 536 nm, respectively. For the assay, approximately 5 × 10⁴ cancer cells were plated in 24-well plates, incubated for 24 h, and treated with the selected compounds for 48 h at their respective IC_50_ and IC_80_ concentrations.

After treatment, the culture medium was refreshed through the addition of fresh medium containing DHR to a final concentration of 5 µM. Cells were incubated for 1 h at 37 °C in an atmosphere of 5% CO_2_ and 95% humidity, then washed and resuspended in PBS with 5 µg/mL PI. The fluorescence intensity of Rh123 was then measured using a FACScan (fluorescence-activated cell sorter) flow cytometer. A total of 10 × 10^3^ cells were analysed per sample and experiments were performed in triplicate with two replicates per assay.

#### 4.2.7. Statistical ANALYSIS

Experimental cytotoxicity data were fitted to a sigmoidal function (y = ymax/(x/a)^-b^) via non-linear regression. IC_20_, IC_50_, and IC_80_ values (concentrations causing 80%, 50%, and 20% inhibition of cell viability, respectively) were obtained via interpolation. These analyses were performed using SigmaPlot^®^ version 12.5 software. All data presented are representative of at least two independent experiments conducted in triplicate. All quantitative data were summarised as mean ± standard deviation (SD).

## 5. Conclusions

In conclusion, in this study we successfully developed the semi-synthesis of six novel *ent*-kaurene derivatives, expanding the structural diversity of this important class of compounds. Among these derivatives, compounds **12** and **13** were subjected to a detailed evaluation of their anticancer potential in direct comparison with their precursor, compound **7**.

Our results showed a significant increase in anticancer activity for derivatives **12** and **13**, demonstrating their improved cytotoxic efficacy compared to compound **7**. This highlights the value of structural modifications in optimising bioactivity and positions these derivatives as promising candidates for further development as anticancer agents. The comparative analysis of the tested compounds revealed that compound **13** exhibited a broad spectrum of activity across cell lines, making it suitable for cancers with deregulated intrinsic apoptotic pathways. Additionally, the selective induction of apoptosis and the absence of significant necrosis underscore the potential of compounds **12** and **13** as anticancer agents with selective and potent apoptotic mechanisms. In contrast to the necrotic pathways, apoptosis minimises inflammation and preserves the tumour microenvironment, making it a preferred mechanism in cancer therapy. Further studies are essential to optimise the efficacy of these derivatives and extend their clinical applicability, offering hope for novel treatments in oncology.

Overall, this work provides critical insights into the structure–activity relationships of the *ent*-kaurene derivatives, paving the way for future explorations in the field of anticancer drug discovery. Future studies are required to determine the full molecular mechanism activated by these compounds and to extend their clinical applicability, offering hope for novel treatments in oncology.

## Figures and Tables

**Table 1:** Effect of *ent*-kaurene diterpenoids on the viability of tumour cell lines HT29, HepG2, and B16-F10 after 72 h of treatment. IC_20_, IC_50_, and IC_80_ values are presented as mean ± standard deviation for three replicates. The ratio between the IC_50_ of compound 7 (precursor) and that of the other compounds (#) is also provided.

Cell Line	Compound	IC_20_ (µM)	IC_50_ (µM)	IC_80_ (µM)	IC_50_ 7/IC_50_ #
HT29	**2**	1.90 ± 1.12	10.08 ± 2.69	50.55 ± 4.37	7.46
	**7**	71.82 ± 0.27	75.27 ± 0.90	79.47 ± 2.53	1.00
	**8**	83.57 ± 4.89	111.72 ± 3.39	148.32 ± 2.67	0.67
	**9**	92.42 ± 2.27	153.83 ± 0.71	202.16 ± 1.70	0.49
	**11**	49.71 ± 3.29	74.96 ± 5.91	114.91 ± 11.43	1.00
	**12**	27.28 ± 8.36	45.37 ± 5.34	85.58 ± 27.54	1.66
	**13**	0.73 ± 0.08	2.71 ± 0.23	8.43 ± 2.61	27.76
HepG2	**2**	39.15 ± 15.37	70.22 ± 9.92	125.00 ± 4.66	1.04
	**7**	71.38 ± 0.66	72.84 ± 0.29	74.30 ± 0.15	1.00
	**8**	66.06 ± 12.57	107.69 ± 6.08	166.59 ± 7.72	0.68
	**9**	48.02 ± 7.57	101.49 ± 7.65	171.30 ± 6.00	0.72
	**11**	31.76 ± 4.10	47.26 ± 5.78	72.90 ± 10.87	1.54
	**12**	8.79 ± 9.30	24.43 ± 16.35	81.75 ± 13.64	2.98
	**13**	0.79 ± 0.30	2.12 ± 0.23	5.36 ± 0.86	34.42
B16-F10	**2**	52.32 ± 3.74	79.58 ± 3.58	125.56 ± 5.55	0.41
	**7**	27.40 ± 4.62	32.43 ± 3.28	37.93 ± 2.94	1.00
	**8**	128.36 ± 2.81	145.32 ± 2.76	168.42 ± 3.60	0.22
	**9**	83.33 ± 4.50	107.13 ± 3.95	138.94 ± 4.68	0.30
	**11**	25.04 ± 2.28	42.04 ± 1.55	66.97 ± 1.05	0.77
	**12**	64.26 ± 6.65	94.54 ± 8.33	143.50 ± 9.36	0.34
	**13**	0.79 ± 0.13	2.65 ± 0.13	54.29 ± 4.00	12.26

## Data Availability

The original contributions presented in the study are included in the article/Supplementary Materials, further inquiries can be directed to the corresponding authors.

## Supplementary Materials

The following supporting information can be downloaded at: https://www.mdpi.com/article/10.3390/ijms252313222/s1.
