# Supplementary material for: Semisynthesis and Antitumour Evaluation of Natural Derivatives from ent-Kaurene ent-15α-Angeloyloxykaur-l6-en-3β-ol Isolated from Distichoselinum tenuifolium
Source: Int J Mol Sci. 2024 Dec 9;25(23):13222. doi: 10.3390/ijms252313222 (PMC11642201; doi:10.3390/ijms252313222)
Supplement: Supplementary file 1 [file ijms-25-13222-s001.zip › ijms-3342196-supplementary/Supporting information.docx]

Semisynthesis and Antitumour Evaluation of Natural Derivatives from *ent-*15α-Angeloyloxykaur-l6-en-3β-ol Isolated from *Distichoselinum tenuifolium*.

Yass K. Yasser ^1^, Daniel Gil ^2,3^, Houda Zentar ^1^, María Jesús Durán-Peña ^3^, Belen Prados-Lopez ^1^, Jorge Juárez-Moreno ^1^, José Manuel Botubol-Ares ^3^, Ali Haidour ^2^, Juan Sainz ^1,4,5,6^, Antonio Fernández ^2^, Ramón Alvarez-Manzaneda ^7^, Rachid Chahboun ^2^* and Fernando J. Reyes-Zurita ^1^*

^1^ Department of Biochemistry and Molecular Biology I, Faculty of Sciences, University of Granada, 18071, Granada, Spain.

^2^ Department of Organic Chemistry, Faculty of Sciences, University of Granada, 18071, Granada, Spain.

^3^ Department of Organic Chemistry, Faculty of Sciences, Campus Universitario Río San Pedro, University of Cádiz, 11510, Puerto Real, Cádiz, Spain.

^4^ Genomic Oncology Area, GENYO, Centre for Genomics and Oncological Research: Pfizer/University of Granada/Andalusian Regional Government, PTS, Granada, Spain.

^5^ CIBER Epidemiología y Salud Pública (CIBERESP), Madrid, Spain.

^6^ Instituto de Investigación Biosanitaria IBs.Granada, Granada, Spain

^7^ Área de Química Orgánica, Departamento de Química y Física, Universidad de Almería, 04120, Almería, Spain.

***** Correspondence: rachid@ugr.es (R.C.); ferjes@ugr.es (F.J.R.-Z.); Tel: +34-958-243-252

Table S1: 1HNMR data of compounds **7** and **8**………………………………….......................S2

Table S2: ^13^CNMR data of compounds **7** and **8**…………………………………….…….....….S3

Figure S1 and Figure S2:.............................................................................................................S4

1D spectra of **2** and 1D and 2D spectra of compounds **7** and **8**……………………........…S5-S41

1D spectra of compounds **9, 10, 11, 12** and **13**…………………………………………..S42-S51

High resolution mass spectra of **2**, **7**-**13**……………………………………………...…..S52-S60

**Table S1:** ^1^HNMR data of compounds **7** and **8**

| Position | **7** | **8** |
| --- | --- | --- |
| 1*α* | 1.92, td (13.2, 3.6) | 1.39-1.27, m |
| 1*β* | 0.92, td (13.2, 4.5) | 2.02, m |
| 2a | 1.60, m | 1.67-1.55, m |
| 2b | 1.65, m |  |
| 3 | 3.20, dd (11.3, 5.1) | 3.10, dd (11.3, 5.0) |
| 5 | 0.66, d (11.8, 2.0) | 1.71, dd (12.5, 2.3) |
| 6a | 1.35, tt (12.5, 7.9) | 1.53-1.42, m |
| 6b | 1.57, m | 1.66-1.56, m |
| 7*α* | 1.47, m | 1.26, m |
| 7*β* |  | 2.09-2.00, m |
| 9 | 1.34, d (6.7) | - |
| 9-OH | - | 3.36, br s |
| 11*α* | 1.72, m | 1.54-1.42, m |
| 11*β* | 1.55, m | 2.12, dd (15.1, 5.8) |
| 12*α* | 1.51, m | 1.90-1.80, m |
| 12*β* | 1.69, m | 1.70-1.62, m |
| 13 | 2.69, t (4.6) | 2.70, m |
| 14a | 1.19, ddt (12.0, 5.1, 1.7) | 2.36, d (12.8) |
| 14b | 2.06, d (12.0) | 1.39-1.29, m |
| 15 | 5.26, t (2.5) | 5.41, t (2.8) |
| 17a | 4.90, m | 4.97, dd (3.0, 1.1) |
| 17b | 4.94, dt (2.8, 0.9) | 4.80, dd (2.6, 1.3) |
| 18 | 0.96, s | 0.96, s |
| 19 | 1.05, s | 0.77, s |
| 20 | 0.77, s | 1.15, s |
| COAng-15 |  |  |
| 3´ | 6.12, qq (7.2, 1.5) | 6.23, qq (7.1, 1.5) |
| 4´ | 2.03, dq (7.2, 1.6) | 2.00, dq (7.2, 1.6) |
| 5’ | 1.94, quint (1.5) | 1.92, quint (1.6) |

**Table S2:** ^13^CNMR data of compounds **7** and **8**

| Position | **7** | **8** |
| --- | --- | --- |
| 1 | 39.1, CH_2_ | 30.3, CH_2_ |
| 2 | 27.5, CH_2_ | 28.2, CH_2_ |
| 3 | 79.1, CH | 78.3, CH |
| 4 | 38.9, C | 39.5, C |
| 5 | 54.9, CH | 47.1, CH |
| 6 | 19.7, CH_2_ | 20.6, CH_2_ |
| 7 | 38.93, CH_2_ | 35.5, CH_2_ |
| 8 | 46.1, C | 50.7, C |
| 9 | 48.3, CH | 79.3, C |
| 10 | 38.87, C | 45.1, C |
| 11 | 17.9, CH_2_ | 31.5, CH_2_ |
| 12 | 33.6, CH_2_ | 36.2, CH_2_ |
| 13 | 40.8, CH | 40.1, CH |
| 14 | 36.6, CH_2_ | 37.1, CH_2_ |
| 15 | 81.3, CH | 84.8, CH |
| 16 | 154.0, C | 153.7, C |
| 17 | 106.4, CH_2_ | 105.5, CH_2_ |
| 18 | 28.5, CH_3_ | 29.1, CH_3_ |
| 19 | 15.7, CH_3_ | 16.3, CH_3_ |
| 20 | 17.8, CH_3_ | 19.8, CH_3_ |
| COAng-15 |  |  |
| 1’ | 168.2, C | 166.7, C |
| 2’ | 128.1, C | 127.6, C |
| 3´ | 138.4, CH | 140.5, CH |
| 4´ | 16.0, CH_3_ | 16.1, CH_3_ |
| 5’ | 21.0, CH_3_ | 20.8, CH_3_ |

Figure S1: 1D nOe detected in **7**

Figure S2: 1D nOe detected in **8**

- ^1^H NMR spectrum of compound **7**

- ^1^H NMR spectrum of compound **7**

^13^C NMR spectrum of compound **7**

gCOSY spectrum of compound **7**

gHSQC spectrum of compound **7**

gHMBC spectrum of compound **7**

1D Selective gradient TOCSY of compound **7** (δ 3.20 signal)

1D Selective gradient TOCSY of compound **7** (δ 3.20 signal)

1D Selective gradient TOCSY of compound **7** (δ 2.69 signal)

1D Selective gradient TOCSY of compound **7** (δ 0.66 signal)

nOe1D spectrum of signal at 5.26 ppm (H-15) of compound **7**

nOe1D spectrum of signal at 3.20 ppm (H-3) of compound **7**

nOe1D spectrum of signal at 2.69 ppm (H-13) of compound **7**

. nOe1D spectrum of signal at 1.05 ppm (H_3_-19) of compound **7**

nOe1D spectrum of signal at 0.77 ppm (H_3_-20) of compound **7**

^1^H NMR spectrum of compound **8**

^13^C NMR spectrum of compound **8**

gCOSY spectrum of compound **8** in acetone-*d*_6_

gHSQC spectrum of compound **8** in acetone-*d*_6_

****gHMBC spectrum of compound **8** in acetone-*d*_6_

1D Selective gradient TOCSY of compound **8** (δ 3.10 signal) in acetone-*d*_6_

1D Selective gradient TOCSY of compound **8** (δ 2.36 signal) in acetone-*d*_6_

1D Selective gradient TOCSY of compound **8** (δ 2.70 signal) in acetone-*d*_6_

1D Selective gradient TOCSY of compound **8** (δ 1.71 signal) in acetone-*d*_6_

nOe1D spectrum of signal at 5.42 ppm (H-15) of compound **8** in acetone-*d*_6_

nOe1D spectrum of signal at 3.10 ppm (H-3) of compound **8** in acetone-*d*_6_

nOe1D spectrum of signal at 2.70 ppm (H-13) of compound **8** in acetone-*d*_6_

nOe1D spectrum of signal at 2.36 ppm (H-14a) of compound **8** in acetone-*d*_6_

nOe1D spectrum of signal at 2.12 ppm (H-7) of compound **8** in acetone-*d*_6_

nOe1D spectrum of signal at 1.15 ppm (H-20) of compound **8** in acetone-*d*_6_

nOe1D spectrum of signal at 0.96 ppm (H-18) of compound **8** in acetone-*d*_6_

nOe1D spectrum of signal at 0.77 ppm (H-19) of compound **8** in acetone-*d*_6_

nOe1D spectrum of signal at 3.36 ppm (9-OH) of compound **8** in acetone-*d*_6_

^1^H NMR spectrum of compound **8**

^13^C NMR spectrum of compound **8**
